# Habitual Short Sleep Duration, Diet, and Development of Type 2 Diabetes in Adults

**DOI:** 10.1001/jamanetworkopen.2024.1147

**Published:** 2024-03-05

**Authors:** Diana Aline Nôga, Elisa de Mello e Souza Meth, André Pekkola Pacheco, Xiao Tan, Jonathan Cedernaes, Lieve Thecla van Egmond, Pei Xue, Christian Benedict

**Affiliations:** 1 Department of Pharmaceutical Biosciences, Uppsala University, Sweden; 2 Department of Big Data in Health Science, Zhejiang University School of Public Health, Hangzhou, China; 3 Sir Run Run Shaw Hospital, Zhejiang University School of Medicine, Hangzhou, China; 4 Department of Medical Sciences, Uppsala University, Uppsala, Sweden; 5 Department of Medical Cell Biology, Uppsala University, Uppsala, Sweden; 6 Department of Psychiatry and Psychotherapy, Tübingen Centre for Mental Health, Medical Faculty, University of Tübingen, Tübingen, Germany

## Abstract

**Importance:**

Understanding the interplay between sleep duration, dietary habits, and the risk of developing type 2 diabetes (T2D) is crucial for public health and diabetes prevention strategies.

**Objective:**

To investigate the associations of type of diet and duration of sleep with the development of T2D.

**Design, Setting, and Participants:**

Data derived from the UK Biobank baseline investigation (2006-2010) were analyzed for this cohort study between May 1 and September 30, 2023. The association between sleep duration and healthy dietary patterns with the risk of T2D was investigated during a median (IQR) follow-up of 12.5 (11.8-13.2) years (end of follow-up, September 30, 2021).

**Exposure:**

For the analysis, 247 867 participants were categorized into 4 sleep duration groups: normal (7-8 hours per day), mild short (6 hours per day), moderate short (5 hours per day), and extreme short (3-4 hours per day). Their dietary habits were evaluated based on population-specific consumption of red meat, processed meat, fruits, vegetables, and fish, resulting in a healthy diet score ranging from 0 (unhealthiest) to 5 (healthiest).

**Main Outcomes and Measures:**

Cox proportional hazards regression analysis was used to calculate hazard ratios (HRs) and 95% CIs for the development of T2D across various sleep duration groups and healthy diet scores.

**Results:**

The cohort comprised 247 867 participants with a mean [SD] age of 55.9 [8.1] years, of whom 52.3% were female. During the follow-up, 3.2% of participants were diagnosed with T2D based on hospital registry data. Cox regression analysis, adjusted for confounding variables, indicated a significant increase in the risk of T2D among participants with 5 hours or less of daily sleep. Individuals sleeping 5 hours per day exhibited a 1.16 adjusted HR (95% CI, 1.05-1.28), and individuals sleeping 3 to 4 hours per day exhibited a 1.41 adjusted HR (95% CI, 1.19-1.68) compared with individuals with normal sleep duration. Furthermore, individuals with the healthiest dietary patterns had a reduced risk of T2D (HR, 0.75 [95% CI, 0.63-0.88]). The association between short sleep duration and increased risk of T2D persisted even for individuals following a healthy diet, but there was no multiplicative interaction between sleep duration and healthy diet score.

**Conclusions and Relevance:**

In this cohort study involving UK residents, habitual short sleep duration was associated with increased risk of developing T2D. This association persisted even among participants who maintained a healthy diet. To validate these findings, further longitudinal studies are needed, incorporating repeated measures of sleep (including objective assessments) and dietary habits.

## Key Points

**Question:**

Is there an association between adherence to healthy diet, sleep duration, and risk of developing type 2 diabetes (T2D) in adults?

**Findings:**

This cohort study analyzing data from 247 867 adults in the UK Biobank found that individuals sleeping less than 6 hours daily had a notably higher risk of developing T2D compared with those with 7 to 8 hours of sleep. Despite the association between healthier diets and reduced T2D risk, the increased risk associated with short sleep duration persisted even among adults with healthy eating habits.

**Meaning:**

These findings suggest that adopting a healthy diet may not reduce the risk of developing T2D among those with habitual short sleep duration.

## Teaser

This cohort study examines whether adherence to a healthy diet and duration of daily sleep are associated with the risk of developing type 2 diabetes among adults.

## Introduction

Many people sleep less than 7 hours per day, a condition often termed as short sleep duration. For instance, according to the 2020 Behavioral Risk Factor Surveillance System, 33.2% of US adults were short sleepers.^1^ Prolonged periods of insufficient sleep are associated with various health risks, including an increased risk of type 2 diabetes (T2D). A meta-analysis of prospective studies involving 482 502 participants with follow-up periods spanning from 2.5 to 16.0 years demonstrated that each hour of sleep duration below 7 hours per day was associated with a 1.09-fold likelihood of developing T2D.^2^ Similar patterns are observed when investigating the association between objectively measured sleep duration and T2D in the UK Biobank. Participants with daily sleep duration below 7 to 8 hours demonstrated a hazard ratio (HR) of 1.21 for the development of T2D.^3^ Further support is derived from various experimental studies that demonstrate impaired glucose tolerance test responses and indicators of insulin resistance associated with acute sleep restriction.^4,5,6,7^

Based on current evidence, increasing daily sleep duration to at least 7 hours may reduce the risk of T2D in individuals with insufficient sleep. Nevertheless, challenges in achieving the recommended sleep duration persist, including factors such as work schedules, childcare responsibilities, and economic pressures. Given those constraints, adhering to an otherwise healthy lifestyle may be an alternative approach for mitigating T2D risk among individuals with short sleep duration. For instance, the results of a small experimental study suggest that engaging in high-intensity interval exercise during the daytime may counteract the detrimental effects of sleep restriction on glucose tolerance in humans.^8^ Those findings were reaffirmed by a recent analysis of UK Biobank data, which indicated that individuals with short sleep duration who engaged in regular physical activity exhibited a lower risk of developing T2D.^3^ While the effectiveness of a healthy dietary pattern in lowering the risk of T2D is well-established,^9,10^ the extent to which adherence to such a diet can mitigate the elevated risk of T2D associated with chronic short sleep duration is less clear. This area of research is particularly challenging due to the tendency of short sleep to promote unhealthy food choices.^11,12,13,14^

Previous research provides substantial evidence that short sleep duration adversely affects glucose metabolism.^15^ In contrast, current literature does not offer strong evidence that extended sleep in individuals with normal sleep patterns significantly disrupts glucose regulation. Thus, the association between habitual long sleep duration (often defined as more than 8 or 9 hours per day) and T2D^2^ may not be causally linked.^16^ With this evidence in mind, our research, encompassing 247 867 participants from the UK Biobank cohort, explored the association between self-reported short sleep duration and T2D incidence, particularly considering adherence to a healthy diet. We hypothesized that a healthy dietary pattern would lower the risk of T2D among those with short sleep duration.

## Methods

### Study Population

This cohort study is part of UK Biobank project No. 80513. Data from 247 867 participants 38 to 71 years of age who took part in the baseline visit (scheduled from 2006 to 2010) were available. We applied multiple criteria, including the absence of data on exposure or confounding variables and a T2D diagnosis within 1 year of assessment, to define the final cohort. A detailed summary of this process is available in eFigure 1 in Supplement 1. The assessment of participants’ daily sleep duration and dietary habits was conducted at baseline as part of a touchscreen questionnaire. The UK Biobank study was approved by the North West Multi-Center Research Ethics Committee^17^; all participants provided written informed consent. This study followed the Strengthening the Reporting of Observational Studies in Epidemiology (STROBE) reporting guideline for cohort studies.

### Assessment of Sleep Duration

Based on a response to the touchscreen question “About how many hours sleep do you get in every 24 hours? (please include naps)” completed during the baseline visit, participants who reported a daily sleep duration of 7 to 8 hours were categorized as having normal sleep duration. Short sleep duration was classified as mild short sleep (6 hours), moderate short sleep (5 hours), and extreme short sleep (3-4 hours) for considering the dose-response relationship with T2D.^2^ In line with previous research,^18^ participants with a daily sleep duration of less than 3 hours were not included in the main analysis (eFigure 1 in Supplement 1).

### Healthy Diet Scale Definition

Similar to a previous UK Biobank study,^19^ participants’ adherence to a healthy diet was determined in the present study via responses to an electronic questionnaire. Healthy eating, based on a population-specific median split, included criteria such as fewer than 2 servings of unprocessed red meat products per week (67.3%), fewer than 2 servings of processed meat products per week (39.2%), 4 or more tablespoons of vegetables per day (64.8%), 2 or more pieces of fruit per day (72.7%), and 2 or more servings of fish products per week (52.3%). Each healthy dietary behavior scored 1 point, resulting in a healthy diet score ranging from 0 (unhealthiest) to 5 (healthiest).

### Incident T2D

The outcome of this study was incident T2D, which was ascertained from hospital inpatient records (*International Statistical Classification of Diseases, Tenth Revision*, codes E110-E119). Records were available until September 30, 2021, and detailed procedures can be found in the UK Biobank online resource.^20^

### Statistical Analysis

Data were analyzed for this cohort study between May 1 and September 30, 2023. All analyses were conducted using SPSS, version 28.0.1.0 (IBM; SPSS Inc), and R version, 4.3.2 (R Project for Statistical Computing). Cox proportional hazards regression analysis was used to calculate HRs and 95% CIs for the development of T2D across various sleep duration groups and the healthy diet score. Statistical significance was defined as a 2-sided *P* < .05. Additionally, we assessed multiplicative and additive interactions between sleep duration and the healthy diet score. For additive interactions, we computed the relative excess risk due to interaction, the attributable proportion due to the interaction, and the synergy index by using the interactionR package (version 0.1.7) in R. The time at risk (measured in days) was calculated from the date of the baseline assessment until the occurrence of T2D diagnosis, death, or the conclusion of the follow-up period (September 30, 2021), whichever came first. Proportional hazards assumptions were verified by assessing Kaplan-Meier survival curves.

To enhance the robustness of the crude associations between sleep duration, adherence to healthy dietary patterns, and incident T2D, the adjusted model considered various participant characteristics from the baseline visit. These characteristics encompassed sleep duration, healthy diet score, age, biological sex (female or male), race and ethnicity (African or Caribbean, Asian, White European, or other [including other ethnic group, any other ethnic background, Black or Black British, other Black background, and other White background] because some racial and ethnic groups reportedly have higher rates of T2D than others^21^; self-reported based on predefined categories from the UK Biobank, which were then combined to form the options used), smoking status (never smoked, previous smoker, current smoker), frequency of weekly alcohol intake (not current, less than 3 times a week, 3 or more times a week), antidepressant use (self-reported use of selective serotonin reuptake inhibitor, selective noradrenaline reuptake inhibitor, tricyclic antidepressant, atypical antidepressant, or monoamine oxidase inhibitor), assessment center region (England, Scotland, or Wales), body mass index, systolic blood pressure (automated reading taken at baseline), socioeconomic status (measured by the Townsend index), educational level (no qualification, university degree, and any other qualification), insomnia symptoms frequency (never or rarely; sometimes; and usually), and physical activity level (categorized as low, moderate, and high levels as defined by the International Physical Activity Questionnaire^22^).

To evaluate the potential competing risk of all-cause death, we computed the Fine-Gray subdistribution hazard by using the cmprsk R package (version 2.2-11). Recommended sleep duration by organizations such as the US Sleep Foundation for adults aged 18 and older is 7 to 9 hours daily.^23^ Consequently, we conducted an additional sensitivity analysis, designating 7 to 9 hours of daily sleep duration as the reference category (eFigure 1 in Supplement 1). Furthermore, we explored the association between short sleep duration and increased T2D risk, considering adherence to individual healthy eating habits. To mitigate bias from inverse causation, we reran the analysis excluding individuals who developed T2D within 5 years after their assessment visit. Finally, the primary analysis was repeated, excluding participants with prediabetes at baseline (hemoglobin A_1c_ [HbA_1c_] levels, 39-47 mmol/mol or 5.7%-6.5% of total hemoglobin^24^; to convert from percentage to proportion of total hemoglobin, multiply by 0.01).

## Results

### Cohort Characteristics

The cohort comprised 247 867 participants with a mean (SD) age of 55.9 (8.1) years, of whom 52.3% were females, 47.7% were males, 93.6% identified as White European, 1.7% as Asian, 0.9% as Caribbean or African, and 3.8% as other race or ethnicity. In addition, 75.5% reported normal sleep duration, 19.8% reported mild short sleep duration, 3.9% reported moderate short sleep duration, and 0.8% reported extreme short sleep duration. Additionally, 1.5% attained a healthy diet score of 0, 7.4% scored 1, 17.6% scored 2, 27.5% scored 3, 29.0% scored 4, and 17.0% scored 5 (defined as the healthiest dietary pattern). Additional cohort characteristics can be found in the Table. Cohort characteristics categorized by either sleep duration or healthy diet score are given in eTable 1 and eTable 2 in Supplement 1.

**Table.** Cohort Characteristics

Characteristic	Participants, No. (%)
Participants, total No.	247 867
Time at risk, mean (SD), y	12 (1.8)
Incident T2D cases during follow-up	7905 (3.2)
Age at baseline, mean (SD), y	55.9 (8.1)
Sex	
Female	129 533 (52.3)
Male	118 334 (47.7)
BMI at baseline, mean (SD)	26.6 (3.7)
Systolic blood pressure at baseline, mean (SD), mm Hg,	138 (17)
HbA_1c_ at baseline, mean (SD)	
Percentage of total hemoglobin	5.4 (2.5)
mmol/mol	34.84 (3.73)
Received antidepressant pharmacotherapy at baseline	7011 (2.8)
Townsend Index, mean (SD)	−1.95 (2.44)
Race and ethnicity	
White European	231 966 (93.6)
Asian	4317 (1.7)
Caribbean or African	2287 (0.9)
Other^a^	9297 (3.8)
Region of the assessment center	
England	228 187 (92.1)
Scotland	10 695 (4.3)
Wales	8985 (3.6)
Physical activity level	
Low	44 612 (18.0)
Moderate	102 447 (41.3)
High	100 808 (40.7)
Smoking at baseline	
Current	22 965 (9.3)
Former	84 823 (34.2)
Never	140 079 (56.5)
Alcohol intake frequency	
3 times a week or more	119 843 (48.4)
Less than 3 times a week	113 826 (45.9)
Not current	14 198 (5.7)
Educational level	
University degree	91 890 (37.1)
Any other qualification	126 128 (50. 9)
No qualification	29 849 (12.0)
Insomnia symptom frequency	
Never or rarely	62 769 (25.3)
Sometimes	118 516 (47.8)
Usually	66 582 (26.9)

Abbreviations: BMI, body mass index (calculated as weight in kilograms divided by height in meters squared); HbA_1c_, hemoglobin A_1c_; T2D, type 2 diabetes.

SI conversion factor: To convert HbA_1c_ percentage to proportion of total, multiply by 0.01.

^a^ Other race and ethnicity included other ethnic group, any other ethnic background, Black or Black British, other Black background, and other White background.

### Associations Between Sleep Duration, Adherence to a Healthy Diet, and Incident T2D

**Figure 1.**
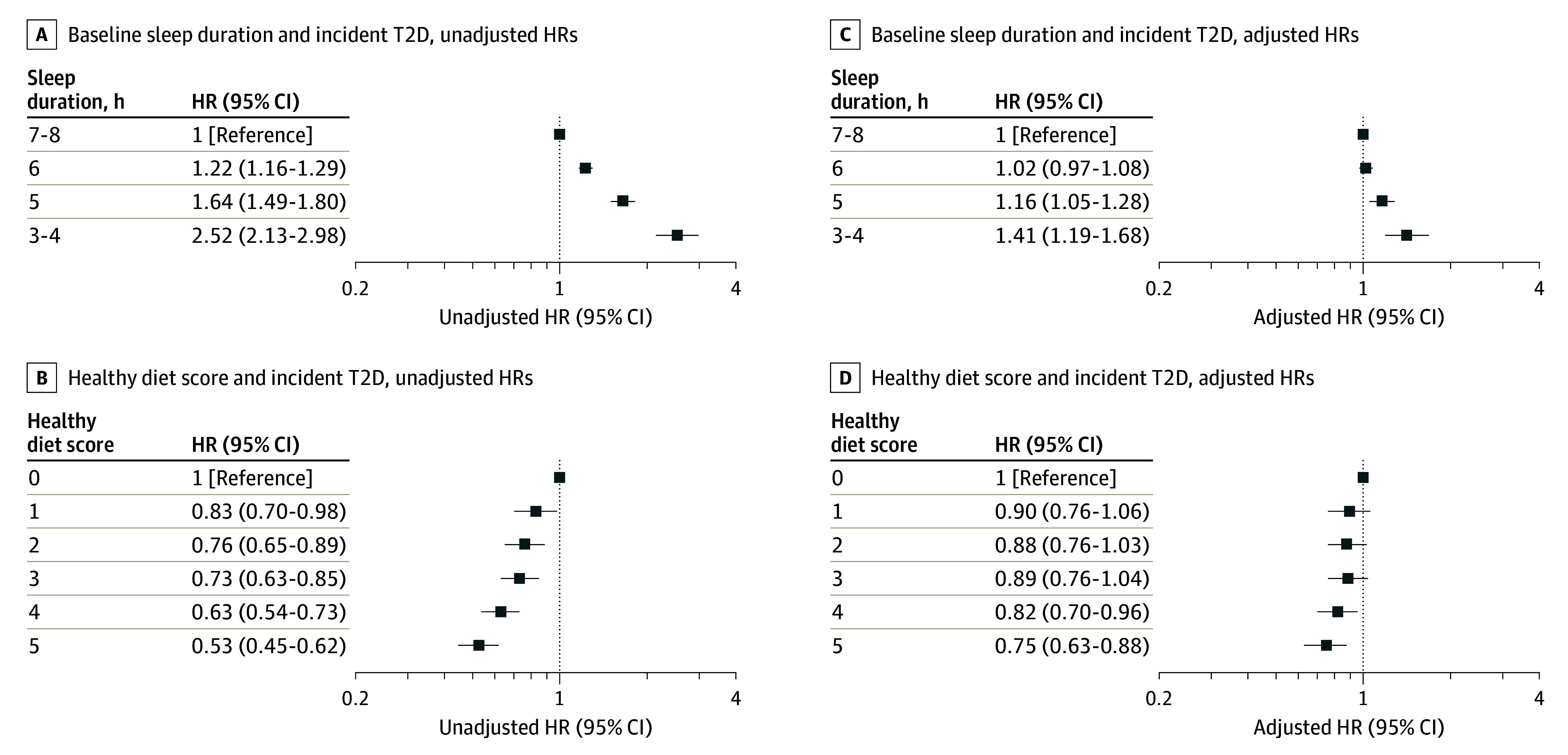
Association of Short Sleep Duration at Baseline and Adherence to Healthy Diet With Incident Type 2 Diabetes (T2D) HR indicates hazard ratio. Reference group in A and C is 7 to 8 hours of sleep duration; B and D, healthy diet score of 0.

**Figure 2.**
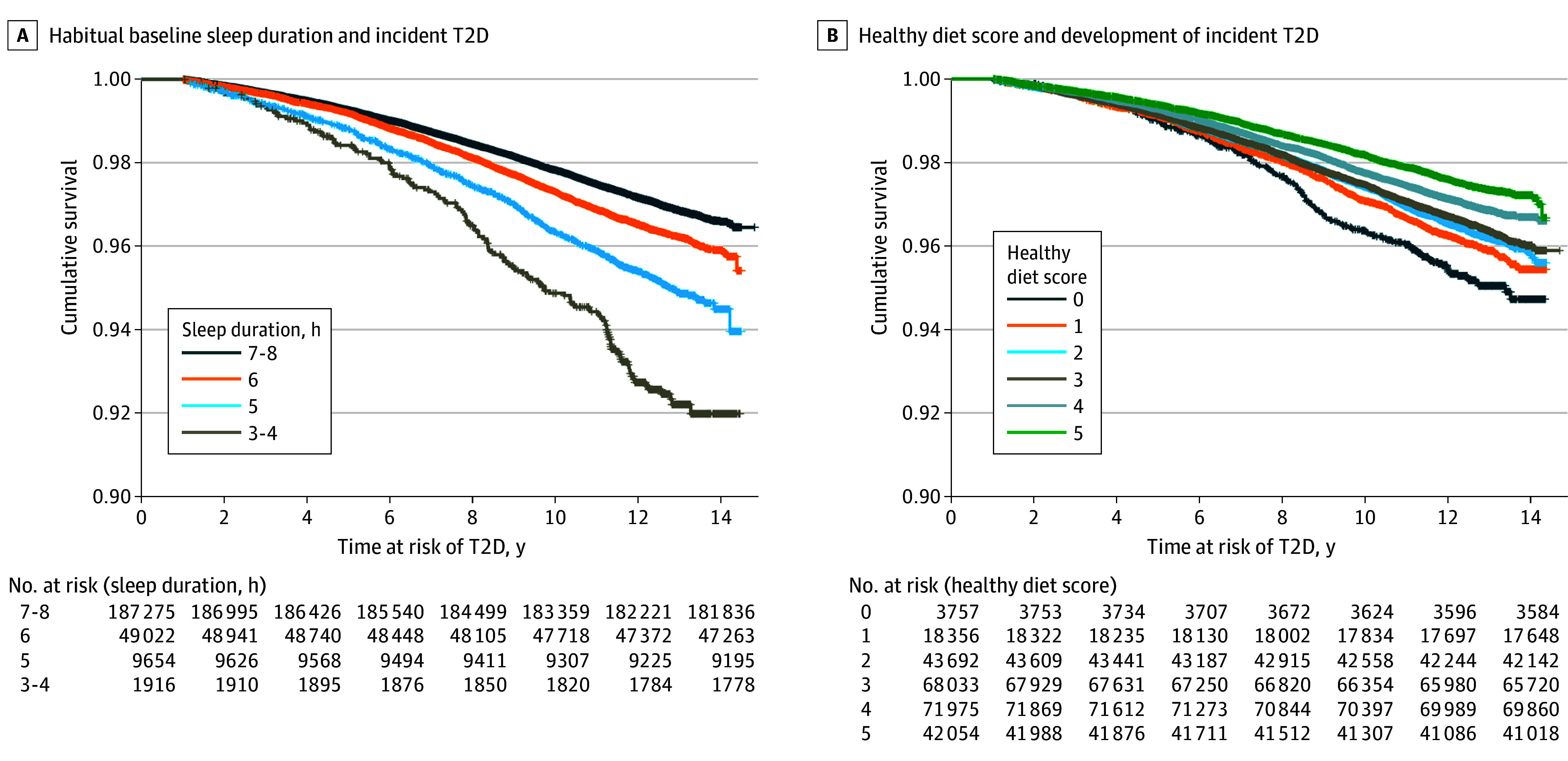
Unadjusted Kaplan-Meier Estimates: Short Sleep Duration, Diet Score, and Type 2 Diabetes (T2D) Risk

The total follow-up time for the investigated cohort was 3 029 282 years at risk, and 7905 participants (3.2%) were diagnosed with T2D during a median (IQR) follow-up of 12.5 (11.8-13.2) years. In comparison with participants reporting normal sleep duration (reference group), participants who reported sleep durations of less than 6 hours per night had greater risk of developing T2D (adjusted HRs,1.16 [95% CI, 1.05-1.28], *P* = .003 for 5 hours; and 1.41 [95% CI, 1.19-1.68], *P* < .001 for 3-4 hours). There was no statistically significant difference between participants who reported normal sleep duration and those who reported 6 hours (adjusted HR, 1.02 [95% CI, 0.97-1.08) (Figure 1). Figure 2 shows Kaplan-Meier curves by sleep duration status.

Participants with a healthy diet score of 4 or 5 exhibited lower risk of developing T2D than those with the least healthy dietary pattern (HR, 0.75 [95% CI, 0.63-0.88], *P* < .001 for 5 points; HR, 0.82 [95% CI, 0.70-0.96], *P* = .01 for 4 points; HR, 0.89 [95% CI, 0.76-1.04], *P* = .13 for 3 points; HR, 0.88 [95% CI, 0.76-1.03], *P* = .12 for 2 points; and HR, 0.90 [95% CI, 0.76-1.06], *P* = .22 for 1 point) (Figure 1). Figure 2 shows Kaplan-Meier plots by healthy diet scale.

**Figure 3.**
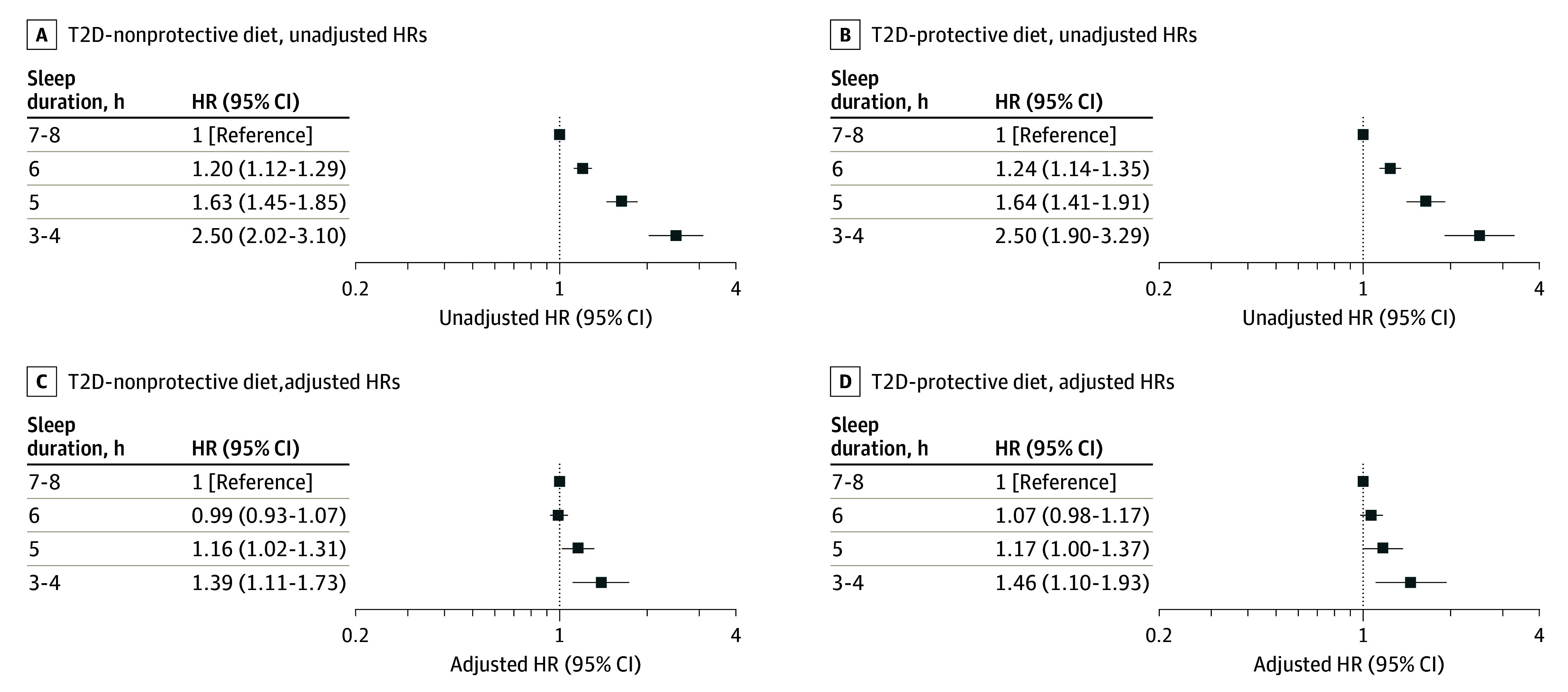
Association Between Short Sleep Duration at Baseline and Incident Type 2 Diabetes (T2D) Stratified by Diet Status HR indicates hazard ratio. Reference group is 7 to 8 hours of sleep duration.

Contrary to our hypothesis, no multiplicative interaction between sleep duration and the healthy diet score was observed, either in the unadjusted (HR [95% CI] range, 0.83-3.02 [0.30-7.20]; *P* = .48) or adjusted (HR [95% CI] range, 0.93-3.49 [0.39-8.34]; *P* = .38) analysis. Considering that sleeping less than 6 hours per day was associated with higher risk, and a healthy diet score of 4 or higher was associated with lower HRs for T2D, we transformed daily sleep duration and the healthy diet score into binary variables to explore a potential additive interaction. Specifically, we categorized daily sleep duration as 6 to 9 hours vs 3 to 5 hours, and the healthy diet score as 4 to 5 points vs 0 to 3 points. There was no significant additive interaction between daily sleep duration and a healthy diet (relative excess risk due to interaction, 0.05 [95% CI, −0.16 to 0.26]; attributable proportion due to the interaction, 0.04 [95% CI, −0.12, 0.19]; synergy index, 1.17 [95% CI, 0.61-2.26]). Figure 3 illustrates the associations between daily sleep duration and T2D incidence during the follow-up, categorized by a T2D-protective dietary pattern (4-5 points) and T2D-nonprotective dietary pattern (0-3 points).

### Sensitivity Analyses

When considering the possible competitive risk of all-cause death, the main results changed for neither sleep (adjusted subdistribution HRs, 1.02 [95% CI, 0.97-1.08], *P* = .48 for mild short sleep duration; 1.16 [95% CI, 1.05-1.28], *P* = .004, for moderate short sleep duration; and 1.41 [95% CI, 1.19-1.69], *P* < .001 for extreme short sleep duration) nor for the healthy diet score (adjusted subdistribution HRs, 0.75 [95% CI, 0.64-0.89], *P* < .001 for 5 points; 0.82 [95% CI, 0.70-0.96], *P* = .02 for 4 points; 0.89 [0.76-1.04], *P* = .15 for 3 points; 0.88 [95% CI, 0.75-1.04], *P* = .13 for 2 points; and 0.90 [95% CI, 0.76-1.07], *P* = .22 for 1 point). When using a daily sleep duration of 7 to 9 hours as the reference category, our results were largely supported (eFigure 2 and eFigure 3 in Supplement 1). These observations persisted even after excluding individuals who developed T2D within the first 5 years of follow-up (eFigure 4 and eFigure 5 in Supplement 1). Examining individual healthy eating habits, we found that a reduced weekly consumption of unprocessed red meat and processed meat was associated with a decreased risk of T2D (eTable 3 in Supplement 1). However, irrespective of whether participants reported high or low consumption of unprocessed red meat and processed meat products, the association between shorter sleep duration and higher HRs for developing T2D remained significant (eTable 4 in Supplement 1).

When excluding participants with prediabetes at baseline, daily sleep durations between 3 and 5 hours remained significantly associated with higher HRs for T2D (eFigure 6 in Supplement 1). None of the healthy diet scores were significantly associated with the risk of T2D (eFigure 6 in Supplement 1). However, when combining participants who scored 4 or 5 on the healthy diet scale as 1 group and participants who scored less as the other group, the risk of developing T2D was lower in the first group in both the unadjusted (HR, 0.77 [95% CI, 0.72-0.83], *P* < .001) and adjusted (HR, 0.81 [95% CI, 0.76-0.88], *P* < .001) analyses. Still, when categorizing participants into those scoring 4 or 5 and those scoring less than 4, short sleep duration remained significantly associated with a higher risk of developing T2D (eFigure 7 in Supplement 1).

## Discussion

This cohort study assessing daily sleep duration, dietary habits, and the risk of T2D among individuals in the UK Biobank cohort 38 to 71 years of age found that habitual short sleep duration was associated with increased risk of developing T2D and that this association persisted even among participants who maintained a healthy diet. Many adults struggle to sleep 7 to 8 hours per day.^1^ As suggested by laboratory studies, a lack of sleep may contribute to the development of T2D through various mechanisms, such as impaired cellular insulin sensitivity,^6^ a skeletal muscle energy metabolism shifted toward nonglucose oxidation,^25^ increased activity of the sympathetic nervous system,^26^ and altered gut microbiota composition.^4,27^ Consequently, the high prevalence of individuals with short sleep duration may contribute to the projected global escalation of T2D prevalence.^28^ Supporting this notion, prospective associations have been observed between short sleep duration and increased risk of T2D. For instance, in the Nurses’ Health Study II and the Whitehall II Study, persistent short sleep duration, defined as either 5.5 hours per day^29^ or 5.5 hours or less per day,^30^ was found to be correlated with a heightened risk of T2D during follow-up.

Recognizing that extending sleep duration may not be a feasible goal for a substantial proportion of individuals with short sleep duration, exploring alternative strategies to mitigate the risk of T2D among them becomes essential. Notably, as suggested by findings from a clinical trial, engaging in high-intensity exercise may mitigate impaired blood glucose control following short sleep.^8^ Consistent with those findings, an analysis of the UK Biobank revealed that individuals with habitual short sleep duration were less likely to develop T2D when regularly engaging in physical activity.^3^ While diets such as the Mediterranean diet, characterized by a high intake of plant-based foods, have been associated with a reduced risk of T2D, habitual eating patterns marked by a high consumption of processed foods, including meat, may have the opposite effect.^31,32,33,34^ However, whether healthy eating habits have the potential to lower T2D risk among habitual short sleepers remains an underexplored research area.

Thus, in the present study, we used data from the UK Biobank baseline assessment, focusing on participants’ weekly consumption of red meat, processed meat, and fish, as well as the daily consumption of vegetables and fruits. This information enabled us to categorize participants into 2 distinct dietary groups: those whose dietary patterns were associated with a lower risk of developing T2D and those whose dietary patterns did not modify the risk of developing T2D. Our findings revealed an elevated T2D risk associated with shorter sleep durations across both dietary groups. These findings, further confirmed in several sensitivity analyses, suggest that healthy dietary habits may not necessarily offset the risk of T2D incurred by habitual short sleep duration.

While our research indeed established a higher risk of T2D associated with short sleep durations, aligning with previous epidemiological and experimental evidence,^2,3,4,5,6,7^ it remains crucial to consider the underlying causes of short sleep duration. For instance, obstructive sleep apnea can lead to premature awakening and insufficient sleep duration.^35^ Notably, a recent analysis indicates that nearly 1 billion individuals worldwide experience sleep-disordered breathing,^36^ with as many as approximately 80% of them likely being unaware of their condition.^37^ Obstructive sleep apnea is known to heighten the risk of insulin resistance and T2D^38,39,40,41^ and may, in part, explain the observed association between short sleep duration and elevated T2D risk. Given this possibility, the efficacy of healthy dietary patterns in mitigating the adverse effects of short sleep on glucose metabolism may be limited if obstructive sleep apnea is coexistent.

### Limitations

Despite the robust nature of our findings—as they remain significant even after adjusting for multiple confounding variables such as participants’ body mass index, age, and weekly physical activity level—a nuanced interpretation is necessary concerning their generalizability. We adopted a method to assess participants’ healthy eating habits similar to a previous UK Biobank study.^19^ However, whether other types of dietary patterns, such as time-restricted eating or the Mediterranean diet, can modify the risk of T2D among individuals with short sleep duration remains unclear. Emerging evidence indicates that such dietary patterns are associated with enhanced blood glucose control and reduced T2D risk.^9,42,43^ Additionally, there may be specific macronutrients or micronutrients, not explored in this study, that could more effectively counteract the adverse metabolic effects induced by sleep loss. Those nutrients may be particularly beneficial for individuals at higher risk of developing conditions such as T2D.^44^ Another limitation of our study is the absence of updated data on follow-up unavailability from the UK Biobank since May 2017. It should also be noted that daily sleep duration and dietary habits were self-reported and only assessed at baseline. This raises concerns about recall bias and the potential variability of those behaviors during the follow-up period. Therefore, to substantiate our findings, additional longitudinal studies are warranted. Those studies should include repeated and objective assessments of sleep and eating habits. Despite our efforts to adjust for a comprehensive range of known confounders, including hypertension, obesity, high HbA_1c_, depression, and various lifestyle factors, the influence of unmeasured variables not captured in our dataset may still play a role in the associations observed between sleep duration, diet, and the risk of T2D. Finally, the majority of our participants were of White ancestry, which may limit the applicability of our results to more diverse populations.

## Conclusions

This cohort study did not yield compelling evidence to support the notion that maintaining a diet characterized by a low consumption of red meat and processed meat products and a high intake of fruits, vegetables, and fish can sufficiently mitigate the risk of developing T2D associated with habitual short sleep duration. However, given the constraints of the current analysis, further research is necessary to explore whether specific dietary patterns, such as time-restricted eating, can counteract or alleviate the adverse metabolic consequences associated with short sleep duration. Future studies exploring the associations among adherence to a healthy diet, sleep duration, and the risk of developing T2D would benefit substantially from including repeated and objective measures of both sleep and dietary habits. Such an approach is essential to unravel the dynamic interplay between these factors in the context of T2D, providing a more comprehensive understanding of their combined association with T2D risk.

## Notes

Supplement 1. **eTable 1.** Cohort Characteristics Split by Sleep Duration Category **eTable 2.** Cohort Characteristics Split by Healthy Diet Score **eTable 3.** Association of Short Sleep Duration and Adherence to Consumption of Individual Food Groups With Incident Type 2 Diabetes Mellitus **eTable 4.** Association Between Short Sleep Duration and Incident Type 2 Diabetes Mellitus Stratified by Adherence to Consumption of Individual Food Groups **eFigure 1.** Final Sample Estimation **eFigure 2.** Association of Short Sleep Duration (Using 7-9 h of Daily Sleep as the Sleep Duration Reference Category) and Adherence to Healthy Diet With Incident Type 2 Diabetes Mellitus **eFigure 3.** Association Between Short Sleep Duration (Using 7-9 h of Daily Sleep as the Sleep Duration Reference Category) and Incident Type 2 Diabetes Mellitus Stratified by Diet Status **eFigure 4.** Association of Short Sleep Duration and Adherence to Healthy Diet With Incident Type 2 Diabetes Mellitus (Without First 5 Years T2D Incidence) **eFigure 5.** Association Between Short Sleep Duration and Incident Type 2 Diabetes Mellitus Stratified by Diet Status (Without First 5 Years T2D Incidence) **eFigure 6.** Association of Short Sleep Duration and Adherence to Healthy Diet With Incident Type 2 Diabetes Mellitus (Without Prediabetic Individuals) **eFigure 7.** Association Between Short Sleep Duration and Incident Type 2 Diabetes Mellitus Stratified by Diet Status (Without Prediabetic Individuals)

Supplement 2.
Data Sharing Statement

## References

[1] Pankowska MM, Lu H, Wheaton AG, . Prevalence and geographic patterns of self-reported short sleep duration among US adults, 2020. Prev Chronic Dis. 2023;20:E53. doi:10.5888/pcd20.220400 37384831 PMC10317035

[2] Shan Z, Ma H, Xie M, . Sleep duration and risk of type 2 diabetes: a meta-analysis of prospective studies. Diabetes Care. 2015;38(3):529-537. doi:10.2337/dc14-2073 25715415

[3] Jin X, Chen Y, Feng H, . Association of accelerometer-measured sleep duration and different intensities of physical activity with incident type 2 diabetes in a population-based cohort study. J Sport Health Sci. 2023;S2095-2546(23)00021-2. doi:10.1016/j.jshs.2023.03.00136871624

[4] Benedict C, Vogel H, Jonas W, . Gut microbiota and glucometabolic alterations in response to recurrent partial sleep deprivation in normal-weight young individuals. Mol Metab. 2016;5(12):1175-1186. doi:10.1016/j.molmet.2016.10.003 27900260 PMC5123208

[5] Cedernaes J, Lampola L, Axelsson EK, . A single night of partial sleep loss impairs fasting insulin sensitivity but does not affect cephalic phase insulin release in young men. J Sleep Res. 2016;25(1):5-10. doi:10.1111/jsr.12340 26361380

[6] Broussard JL, Ehrmann DA, Van Cauter E, Tasali E, Brady MJ. Impaired insulin signaling in human adipocytes after experimental sleep restriction: a randomized, crossover study. Ann Intern Med. 2012;157(8):549-557. doi:10.7326/0003-4819-157-8-201210160-00005 23070488 PMC4435718

[7] Buxton OM, Pavlova M, Reid EW, Wang W, Simonson DC, Adler GK. Sleep restriction for 1 week reduces insulin sensitivity in healthy men. Diabetes. 2010;59(9):2126-2133. doi:10.2337/db09-0699 20585000 PMC2927933

[8] Saner NJ, Lee MJ, Kuang J, . Exercise mitigates sleep-loss-induced changes in glucose tolerance, mitochondrial function, sarcoplasmic protein synthesis, and diurnal rhythms. Mol Metab. 2021;43:101110. doi:10.1016/j.molmet.2020.101110 33137489 PMC7704425

[9] Koloverou E, Esposito K, Giugliano D, Panagiotakos D. The effect of Mediterranean diet on the development of type 2 diabetes mellitus: a meta-analysis of 10 prospective studies and 136,846 participants. Metabolism. 2014;63(7):903-911. doi:10.1016/j.metabol.2014.04.010 24931280

[10] Neuenschwander M, Ballon A, Weber KS, . Role of diet in type 2 diabetes incidence: umbrella review of meta-analyses of prospective observational studies. BMJ. 2019;366:l2368. doi:10.1136/bmj.l2368 31270064 PMC6607211

[11] St-Onge MP, Cherta-Murillo A, Darimont C, Mantantzis K, Martin FP, Owen L. The interrelationship between sleep, diet, and glucose metabolism. Sleep Med Rev. 2023;69:101788. doi:10.1016/j.smrv.2023.101788 37156196 PMC10247426

[12] St-Onge MP. Sleep-obesity relation: underlying mechanisms and consequences for treatment. Obes Rev. 2017;18(suppl 1):34-39. doi:10.1111/obr.12499 28164452 PMC13098705

[13] St-Onge MP, Wolfe S, Sy M, Shechter A, Hirsch J. Sleep restriction increases the neuronal response to unhealthy food in normal-weight individuals. Int J Obes (Lond). 2014;38(3):411-416. doi:10.1038/ijo.2013.114 23779051 PMC3883872

[14] St-Onge MP, McReynolds A, Trivedi ZB, Roberts AL, Sy M, Hirsch J. Sleep restriction leads to increased activation of brain regions sensitive to food stimuli. Am J Clin Nutr. 2012;95(4):818-824. doi:10.3945/ajcn.111.027383 22357722 PMC3302360

[15] Sondrup N, Termannsen AD, Eriksen JN, . Effects of sleep manipulation on markers of insulin sensitivity: a systematic review and meta-analysis of randomized controlled trials. Sleep Med Rev. 2022;62:101594. doi:10.1016/j.smrv.2022.101594 35189549

[16] Tan X, Chapman CD, Cedernaes J, Benedict C. Association between long sleep duration and increased risk of obesity and type 2 diabetes: a review of possible mechanisms. Sleep Med Rev. 2018;40:127-134. doi:10.1016/j.smrv.2017.11.001 29233612

[17] National Research Ethics Service. UK Biobank ethical approval. Accessed May 1, 2023. https://www.ukbiobank.ac.uk/media/cs1h15s3/rtb-nwrec-application-and-approval-2011.pdf

[18] Xue P, Merikanto I, Chung F, . Persistent short nighttime sleep duration is associated with a greater post-COVID risk in fully mRNA-vaccinated individuals. Transl Psychiatry. 2023;13(1):32. doi:10.1038/s41398-023-02334-4 36726008 PMC9890416

[19] Feng H, Yang L, Liang YY, . Associations of timing of physical activity with all-cause and cause-specific mortality in a prospective cohort study. Nat Commun. 2023;14(1):930. doi:10.1038/s41467-023-36546-5 36805455 PMC9938683

[20] UK Biobank. Homepage for the UK Biobank. Accessed May 1, 2023. https://www.ukbiobank.ac.uk

[21] Shai I, Jiang R, Manson JE, et al. Ethnicity, obesity, and risk of type 2 diabetes in women: a 20-year follow-up study. Diabetes Care. 2006;29(7):1585-1590. doi:10.2337/dc06-0057 16801583

[22] Craig CL, Marshall AL, Sjöström M, . International physical activity questionnaire: 12-country reliability and validity. Med Sci Sports Exerc. 2003;35(8):1381-1395. doi:10.1249/01.MSS.0000078924.61453.FB 12900694

[23] Hirshkowitz M, Whiton K, Albert SM, . National Sleep Foundation’s updated sleep duration recommendations: final report. Sleep Health. 2015;1(4):233-243. doi:10.1016/j.sleh.2015.10.004 29073398

[24] American Diabetes Association. 2. Classification and diagnosis of diabetes: standards of medical care in diabetes-2021. Diabetes Care. 2021;44(suppl 1):S15-S33. doi:10.2337/dc21-S002 33298413

[25] Cedernaes J, Schönke M, Westholm JO, . Acute sleep loss results in tissue-specific alterations in genome-wide DNA methylation state and metabolic fuel utilization in humans. Sci Adv. 2018;4(8):eaar8590. doi:10.1126/sciadv.aar8590 30140739 PMC6105229

[26] Spiegel K, Leproult R, Van Cauter E. Impact of sleep debt on metabolic and endocrine function. Lancet. 1999;354(9188):1435-1439. doi:10.1016/S0140-6736(99)01376-8 10543671

[27] Arnoriaga-Rodríguez M, Leal Y, Mayneris-Perxachs J, . Gut microbiota composition and functionality are associated with REM sleep duration and continuous glucose levels. J Clin Endocrinol Metab. 2023;108(11):2931-2939. doi:10.1210/clinem/dgad258 37159524

[28] Reutrakul S, Van Cauter E. Sleep influences on obesity, insulin resistance, and risk of type 2 diabetes. Metabolism. 2018;84:56-66. doi:10.1016/j.metabol.2018.02.010 29510179

[29] Baden MY, Hu FB, Vetter C, Schernhammer E, Redline S, Huang T. Sleep duration patterns in early to middle adulthood and subsequent risk of type 2 diabetes in women. Diabetes Care. 2020;43(6):1219-1226. doi:10.2337/dc19-2371 32209646 PMC7245349

[30] Ferrie JE, Kivimäki M, Akbaraly TN, . Change in sleep duration and type 2 diabetes: the Whitehall II study. Diabetes Care. 2015;38(8):1467-1472. doi:10.2337/dc15-0186 26068863 PMC4512137

[31] Ahmad S, Demler OV, Sun Q, . Association of the Mediterranean diet with onset of diabetes in the Women’s Health Study. JAMA Netw Open. 2020;3(11):e2025466. doi:10.1001/jamanetworkopen.2020.25466 33211107 PMC7677766

[32] de Koning L, Chiuve SE, Fung TT, Willett WC, Rimm EB, Hu FB. Diet-quality scores and the risk of type 2 diabetes in men. Diabetes Care. 2011;34(5):1150-1156. doi:10.2337/dc10-2352 21464460 PMC3114491

[33] Shi W, Huang X, Schooling CM, Zhao JV. Red meat consumption, cardiovascular diseases, and diabetes: a systematic review and meta-analysis. Eur Heart J. 2023;44(28):2626-2635. doi:10.1093/eurheartj/ehad336 37264855

[34] Wang PY, Fang JC, Gao ZH, Zhang C, Xie SY. Higher intake of fruits, vegetables or their fiber reduces the risk of type 2 diabetes: a meta-analysis. J Diabetes Investig. 2016;7(1):56-69. doi:10.1111/jdi.12376 26816602 PMC4718092

[35] Eckert DJ, White DP, Jordan AS, Malhotra A, Wellman A. Defining phenotypic causes of obstructive sleep apnea. Identification of novel therapeutic targets. Am J Respir Crit Care Med. 2013;188(8):996-1004. doi:10.1164/rccm.201303-0448OC 23721582 PMC3826282

[36] Benjafield AV, Ayas NT, Eastwood PR, . Estimation of the global prevalence and burden of obstructive sleep apnoea: a literature-based analysis. Lancet Respir Med. 2019;7(8):687-698. doi:10.1016/S2213-2600(19)30198-5 31300334 PMC7007763

[37] Finkel KJ, Searleman AC, Tymkew H, . Prevalence of undiagnosed obstructive sleep apnea among adult surgical patients in an academic medical center. Sleep Med. 2009;10(7):753-758. doi:10.1016/j.sleep.2008.08.007 19186102

[38] Mokhlesi B, Tjaden AH, Temple KA, ; RISE Consortium. Obstructive sleep apnea, glucose tolerance, and β-cell function in adults with prediabetes or untreated type 2 diabetes in the Restoring Insulin Secretion (RISE) study. Diabetes Care. 2021;44(4):993-1001. doi:10.2337/dc20-2127 33547205 PMC7985427

[39] Mokhlesi B, Grimaldi D, Beccuti G, . Effect of one week of 8-hour nightly continuous positive airway pressure treatment of obstructive sleep apnea on glycemic control in type 2 diabetes: a proof-of-concept study. Am J Respir Crit Care Med. 2016;194(4):516-519. doi:10.1164/rccm.201602-0396LE 27525461 PMC5003331

[40] Grimaldi D, Beccuti G, Touma C, Van Cauter E, Mokhlesi B. Association of obstructive sleep apnea in rapid eye movement sleep with reduced glycemic control in type 2 diabetes: therapeutic implications. Diabetes Care. 2014;37(2):355-363. doi:10.2337/dc13-0933 24101701 PMC3898763

[41] Tasali E, Mokhlesi B, Van Cauter E. Obstructive sleep apnea and type 2 diabetes: interacting epidemics. Chest. 2008;133(2):496-506. doi:10.1378/chest.07-0828 18252916

[42] Cienfuegos S, McStay M, Gabel K, Varady KA. Time restricted eating for the prevention of type 2 diabetes. J Physiol. 2022;600(5):1253-1264. doi:10.1113/JP281101 34418079

[43] Haganes KL, Silva CP, Eyjólfsdóttir SK, . Time-restricted eating and exercise training improve HbA1c and body composition in women with overweight/obesity: a randomized controlled trial. Cell Metab. 2022;34(10):1457-1471.e4. doi:10.1016/j.cmet.2022.09.003 36198292

[44] Gao H, Geng T, Huang T, Zhao Q. Fish oil supplementation and insulin sensitivity: a systematic review and meta-analysis. Lipids Health Dis. 2017;16(1):131. doi:10.1186/s12944-017-0528-0 28673352 PMC5496233

