# Supplementary material for: Habitual Short Sleep Duration, Diet, and Development of Type 2 Diabetes in Adults
Source: JAMA Netw Open. 2024 Mar 5;7(3):e241147. doi: 10.1001/jamanetworkopen.2024.1147 (PMC10915681; doi:10.1001/jamanetworkopen.2024.1147)
Supplement: Supplement 1. — eTable 1. Cohort Characteristics Split by Sleep Duration Category eTable 2. Cohort Characteristics Split by Healthy Diet Score eTable 3. Association of Short Sleep Duration and Adherence to Consumption of Individual Food Groups With Incident Type 2 Diabetes Mellitus eTable 4. Association Between Short Sleep Duration and Incident Type 2 Diabetes Mellitus Stratified by Adherence to Consumption of Individual Food Groups eFigure 1. Final Sample Estimation eFigure 2. Association of Short Sleep Duration (Using 7-9 h of Daily Sleep as the Sleep Duration Reference Category) and Adherence to Healthy Diet With Incident Type 2 Diabetes Mellitus eFigure 3. Association Between Short Sleep Duration (Using 7-9 h of Daily Sleep as the Sleep Duration Reference Category) and Incident Type 2 Diabetes Mellitus Stratified by Diet Status eFigure 4. Association of Short Sleep Duration and Adherence to Healthy Diet With Incident Type 2 Diabetes Mellitus (Without First 5 Years T2D Incidence) eFigure 5. Association Between Short Sleep Duration and Incident Type 2 Diabetes Mellitus Stratified by Diet Status (Without First 5 Years T2D Incidence) eFigure 6. Association of Short Sleep Duration and Adherence to Healthy Diet With Incident Type 2 Diabetes Mellitus (Without Prediabetic Individuals) eFigure 7. Association Between Short Sleep Duration and Incident Type 2 Diabetes Mellitus Stratified by Diet Status (Without Prediabetic Individuals) [file jamanetwopen-e241147-s001.pdf]

# Supplementary Online Content

Nôga DA, de Mello e Souza Meth E, Pacheco AP, et al. Habitual short sleep duration, diet, and development of type 2 diabetes. *JAMA Netw Open*. 2024;7(3):e241147. doi:10.1001/jamanetworkopen.2024.1147

**eTable 1.** Cohort Characteristics Split by Sleep Duration Category
**eTable 2.** Cohort Characteristics Split by Healthy Diet Score
**eTable 3.** Association of Short Sleep Duration and Adherence to Consumption of Individual Food Groups With Incident Type 2 Diabetes Mellitus
**eTable 4.** Association Between Short Sleep Duration and Incident Type 2 Diabetes Mellitus Stratified by Adherence to Consumption of Individual Food Groups
**eFigure 1.** Final Sample Estimation
**eFigure 2.** Association of Short Sleep Duration (Using 7-9 h of Daily Sleep as the Sleep Duration Reference Category) and Adherence to Healthy Diet With Incident Type 2 Diabetes Mellitus
**eFigure 3.** Association Between Short Sleep Duration (Using 7-9 h of Daily Sleep as the Sleep Duration Reference Category) and Incident Type 2 Diabetes Mellitus Stratified by Diet Status
**eFigure 4.** Association of Short Sleep Duration and Adherence to Healthy Diet With Incident Type 2 Diabetes Mellitus (Without First 5 Years T2DM Incidence)
**eFigure 5.** Association Between Short Sleep Duration and Incident Type 2 Diabetes Mellitus Stratified by Diet Status (Without First 5 Years T2DM Incidence)
**eFigure 6.** Association of Short Sleep Duration and Adherence to Healthy Diet With Incident Type 2 Diabetes Mellitus (Without Prediabetic Individuals)
**eFigure 7.** Association Between Short Sleep Duration and Incident Type 2 Diabetes Mellitus Stratified by Diet Status (Without Prediabetic Individuals)

This supplementary material has been provided by the authors to give readers additional information about their work.

© 2024 Nôga DA et al. *JAMA Network Open*.

## eTable 1. Cohort Characteristics Split by Sleep Duration Category

| Characteristic | Daily Sleep Duration in Hours, n (%) | | | |
|---|---|---|---|---|
| | 7-8 | 6 | 5 | 3-4 |
| Participants | 187 275 (75.5) | 49 022 (19.8) | 9 654 (3.9) | 1 916 (0.8) |
| Time at risk, days, mean ± SD | 4 472 ± 644 | 4 447 ± 672 | 4 400 ± 748 | 4356 ± 788 |
| Incident T2DM cases during follow-up | 5 544 (3.0) | 1 763 (3.6) | 460 (4.8) | 138 (7.2) |
| Age at baseline, years, mean ± SD | 55.9 ± 8.1 | 55.9 ±7.8 | 56.5 ± 7.8 | 56.7 ± 7.7 |
| Sex | | | | |
| *Female* | 98 412 (52.5) | 24 711 (50.4) | 5 314 (55.0) | 1 096 (57.2) |
| *Male* | 88 863 (45.5) | 24 311 (49.6) | 4 340 (45.0) | 820 (42.8) |
| BMI at baseline, kg/m$^2$, mean ± SD | 26.4 ± 3.6 | 26.9 ±3.7 | 27.2 ± 3.8 | 27.4 ± 4.0 |
| Systolic blood pressure at baseline, mmHg, mean ± SD | 137.6 ± 17.1 | 137.7 ± 16.7 | 138.4 ±16.9 | 137.8 ± 16.9 |
| HbA1c at baseline, mmol/mol, mean ± SD | 34.8 ± 3.7 | 35.1 ± 3.7 | 35.3 ± 3.8 | 35.6 ± 3.8 |
| On antidepressant pharmacotherapy at baseline | 5 105 (2.7) | 1 385 (2.8) | 406 (4.2) | 115 (6.0) |
| Townsend Index, mean ± SD | -2.02 ± 2.41 | -1.78 ± 2.49 | -1.53 ± 2.56 | -1.04 ± 2.72 |
| Ethnicity | | | | |
| *White European* | 176 202 (94.1) | 45 224 (92.3) | 8 843 (91.6) | 1 697 (88.6) |
| *Asian* | 3 000 (1.6) | 1 047 (2.1) | 202 (2.1) | 68 (3.5) |
| *Caribbean or African* | 1 206 (0.6) | 756 (1.5) | 262 (2.7) | 63 (3.3) |
| *Others* | 6 867 (3.7) | 1 995 (4.1) | 347 (3.6) | 88 (4.6) |
| Region of the assessment center | | | | |
| *England* | 172 316 (92.0) | 45 207 (92.2) | 8 883 (92.0) | 1781 (93.0) |
| *Scotland* | 7 936 (4.2) | 2 220 (4.5) | 459 (4.8) | 80 (4.2) |
| *Wales* | 7 023 (3.8) | 1 595 (3.3) | 312 (3.2) | 55 (2.9) |
| Physical activity level | | | | |
| *Low* | 32 880 (17.6) | 9 325 (19.0) | 1 955 (20.3) | 452 (23.6) |
| *Moderate* | 78 560 (41.9) | 19 596 (40.0) | 3 634 (37.7) | 657 (34.3) |
| *High* | 75 835 (40.5) | 20 101 (41.0) | 4 065 (42.0) | 807 (42.1) |
| Smoking at baseline | | | | |
| *Never* | 107 261 (57.3) | 26 669 (54.4) | 5 120 (53.0) | 1 029 (53.7) |
| *Former* | 63 744 (34.0) | 17 143 (35.0) | 3 338 (34.6) | 598 (31.2) |
| *Current* | 16 270 (8.7) | 5 210 (10.6) | 1 196 (12.4) | 289 (15.1) |
| Alcohol intake frequency | | | | |
| *Not current* | 9 981 (5.3) | 3 063 (6.2) | 887 (9.2) | 267 (13.9) |
| *Less than 3 times a week* | 84 880 (45.3) | 23 178 (47.3) | 4 784 (49.5) | 984 (51.4) |
| *3 times a week or more* | 92 414 (49.4) | 22 781 (46.5) | 3 983 (41.3) | 665 (34.7) |
| Educational level | | | | |
| *No qualification* | 72 141 (38.5) | 16 816 (34.3) | 2 559 (26.5) | 374 (19.5) |
| *Any other qualification* | 94 275 (50.3) | 25 590 (52.2) | 5 250 (54.4) | 1 013 (52.9) |
| *University degree* | 20 859 (11.2) | 6 616 (13.5) | 1 845 (19.1) | 529 (27.6) |
| Insomnia symptoms frequency | | | | |
| *Never/rarely* | 53 112 (28.4) | 8 626 (17.6) | 946 (9.8) | 85 (4.4) |
| *Sometimes* | 97 157 (51.8) | 19 159 (39.1) | 2 035 (21.1) | 165 (8.6) |
| *Usually* | 37 006 (19.8) | 21 237 (43.3) | 6 673 (69.1) | 1 666 (87.0) |

*Abbreviations: BMI, body mass index; HbA1c, hemoglobin A1c.*

© 2024 Nôga DA et al. *JAMA Network Open*.

## eTable 2. Cohort Characteristics Split by Healthy Diet Score

| Characteristic | Healthy Diet Score (0 = Least Healthy; 5 = Most Healthy), n (%) | | | | | |
|---|---|---|---|---|---|---|
| | 0 | 1 | 2 | 3 | 4 | 5 |
| Participants | 3 757 (1.5) | 18 356 (7.4) | 43 692 (17.6) | 68 033 (27.5) | 71 975 (29.0) | 42 054 (17.0) |
| Time at risk, days, mean ± SD | 4 414 ±739 | 4 438 ± 702 | 4 452 ± 676 | 4 457 ± 672 | 4 474 ± 634 | 4 483 ± 613 |
| Incident T2DM cases during follow-up | 173 (4.6) | 708 (3.9) | 1 554 (3.6) | 2 315 (3.4) | 2 116 (2.9) | 1 039 (2.5) |
| Age at baseline, years, mean ± SD | 53.8 ± 8.1 | 54.1 ± 8.2 | 54.8 ± 8.2 | 55.7 ± 8.1 | 56.4 ± 8.0 | 57.2 ± 7.7 |
| Sex | | | | | | |
| *Female* | 836 (22.3) | 5 442 (29.6) | 17 610 (40.3) | 33 969 (49.9) | 42 911 (59.6) | 28 765 (68.4) |
| *Male* | 2 921 (77.7) | 12 921 (70.4) | 26 077 (59.7) | 34 062 (50.1) | 29 065 (40.4) | 13 288 (31.6) |
| BMI at baseline, kg/m$^2$, mean ± SD | 27.3 ± 3.7 | 27.1 ± 3.7 | 26.9 ± 3.7 | 26.6 ± 3.6 | 26.4 ± 3.7 | 26.1 ± 3.6 |
| Systolic blood pressure at baseline, mmHg, mean ± SD | 138.6 ± 16.4 | 138.1 ± 16.4 | 137.6 ± 16.8 | 137.7 ± 17.0 | 137.5 ± 17.2 | 137.4 ± 17.3 |
| HbA1c at baseline, mmol/mol, mean ± SD | 35.1 ± 3.9 | 35.0 ± 3.9 | 34.9 ± 3.8 | 34.8 ± 3.8 | 34.8 ± 3.7 | 34.8 ± 3.7 |
| On antidepressant pharmacotherapy at baseline | 94 (2.5) | 445 (2.4) | 1 251 (2.9) | 1 918 (2.8) | 2 093 (2.9) | 1 210 (2.9) |
| Townsend Index, mean ± SD | -1.57 ± 2.56 | -1.80 ± 2.51 | -1.88 ± 2.47 | -1.97 ± 2.43 | -1.99 ± 2.41 | -2.01 ± 2.42 |
| Ethnicity | | | | | | |
| *White European* | 3 559 (94.7) | 17 418 (94.3) | 41 208 (94.3) | 63 791 (93.8) | 66 843 (92.9) | 39 147 (93.0) |
| *Asian* | 46 (1.2) | 245 (1.3) | 651 (1.5) | 1 209 (1.8) | 1 544 (2.1) | 622 (1.5) |
| *Caribbean or African* | 40 (1.1) | 177 (1.0) | 400 (0.9) | 609 (0.9) | 654 (0.9) | 407 (1.0) |
| *Others* | 112 (3.0) | 523 (2.8) | 1 428 (3.3) | 2 422 (3.6) | 2 935 (4.1) | 1 877 (4.5) |
| Region of the assessment center | | | | | | |
| *England* | 3 413 (90.9) | 16 772 (91.4) | 40 097 (91.8) | 62 619 (92.0) | 66 350 (92.2) | 38 936 (92.6) |
| *Scotland* | 143 (3.8) | 780 (4.2) | 1 832 (4.2) | 2 916 (4.3) | 3 161 (4.4) | 1 863 (4.4) |
| *Wales* | 201 (5.3) | 811 (4.4) | 1 758 (4.0) | 2 496 (3.7) | 2 465 (3.4) | 1 254 (3.0) |
| Physical activity level | | | | | | |
| *Low* | 1 024 (27.3) | 4 721 (25.7) | 9 865 (22.5) | 12 802 (18.8) | 10 905 (15.2) | 5 295 (12.6) |
| *Moderate* | 1 478 (39.3) | 7 488 (40.8) | 18 329 (42.0) | 28 746 (42.3) | 29 736 (41.3) | 16 670 (39.6) |
| *High* | 1 255 (33.4) | 6 154 (33.5) | 15 493 (35.5) | 26 483 (38.9) | 31 335 (43.5) | 20 088 (47.8) |
| Smoking at baseline | | | | | | |
| *Never* | 1 781 (47.4) | 9 422 (51.3) | 24 025 (55.0) | 38 760 (57.0) | 41 726 (58.0) | 24 365 (57.9) |
| *Former* | 1 131 (30.1) | 5 848 (31.9) | 14 299 (32.7) | 23 127 (34.0) | 25 168 (35.0) | 15 250 (36.3) |
| *Current* | 845 (22.5) | 3 093 (16.8) | 5 363 (12.3) | 6 144 (9.0) | 5 082 (7.0) | 2 438 (5.8) |
| Alcohol intake frequency | | | | | | |
| *Not current* | 185 (4.9) | 937 (5.1) | 2393 (5.5) | 3 873 (5.7) | 4 366 (6.1) | 2 444 (5.8) |
| *Less than 3 times a week* | 1 461 (38.9) | 7 804 (42.5) | 19 446 (44.5) | 31 044 (45.6) | 33 890 (47.1) | 20 181 (48.0) |
| *3 times a week or more* | 2 111 (56.2) | 9 622 (42.4) | 21 848 (50.0) | 33 114 (48.7) | 33 720 (46.9) | 19 428 (46.2) |

© 2024 Nôga DA et al. *JAMA Network Open*.

| | | | | | | |
|---|---|---|---|---|---|---|
| Educational level, n (%) | | | | | | |
| *No qualification* | 540 (14.4) | 2 480 (13.5) | 5 480 (12.5) | 8 300 (12.2) | 8 245 (11.4) | 4 804 (11.5) |
| *Any other qualification* | 2 083 (55.4) | 10 134 (55.2) | 23 118 (52.9) | 34 757 (51.1) | 35 456 (49.3) | 20 580 (48.9) |
| *University degree* | 1 134 (30.2) | 5 749 (31.3) | 15 089 (34.5) | 24 974 (36.7) | 28 275 (39.3) | 16 669 (39.6) |
| Insomnia symptoms frequency | | | | | | |
| *Never/rarely* | 1 003 (26.7) | 4 844 (26.4) | 11 535 (26.4) | 17 352 (25.5) | 17 914 (24.9) | 10 121 (24.1) |
| *Sometimes* | 1 721 (45.8) | 8 538 (46.5) | 20 498 (46.9) | 32 579 (47.9) | 34 601 (48.1) | 20 579 (48.9) |
| *Usually* | 1 033 (27.5) | 4 981 (27.1) | 11 654 (26.7) | 18 100 (26.6) | 19 461 (27.0) | 11 353 (27.0) |

*Abbreviations*: *BMI, body mass index; HbA1c, hemoglobin A1c.*

© 2024 Nôga DA et al. *JAMA Network Open*.

**eTable 3. Association of Short Sleep Duration and Adherence to Consumption of Individual Food Groups With Incident Type 2 Diabetes Mellitus**

| **Exposure** | **Participants, n** | **T2DM cases[1], %** | **HR [95%-CI]** | |
|---|---|---|---|---|
| | | | **unadjusted** | **adjusted[2]** |
| Sleep duration (hours/day) | | | | |
| *7-8* | 187 275 | 3.0 | 1 | 1 |
| *6* | 49 022 | 3.6 | 1.22 [1.16-1.29] | 1.02 [0.97-1.08] |
| *5* | 9 654 | 4.8 | 1.64 [1.49-1.81] | 1.16 [1.05-1.28] |
| *3-4* | 1 916 | 7.2 | 2.52 [2.12-2.98] | 1.41 [1.19-1.68] |
| | | | | |
| *Weekly consumption of red meat* | | | | |
| > population median | 81 048 | 3.8 | 1 | 1 |
| ≤ population median | 166 819 | 2.9 | 0.75 [0.72-0.79] | 0.90 [0.86-0.94] |
| | | | | |
| *Weekly consumption of processed meat* | | | | |
| > population median | 150 797 | 3.5 | 1 | 1 |
| ≤ population median | 97 070 | 2.7 | 0.75 [0.72-0.79] | 0.95 [0.90-1.0] |
| | | | | |
| *Daily consumption of vegetables* | | | | |
| < population median | 87 378 | 3.3 | 1 | 1 |
| ≥ population median | 160 489 | 3.1 | 0.96 [0.92-1.01] | 0.96 [0.91-1.01] |
| | | | | |
| *Daily consumption of fruits* | | | | |
| < population median | 67 655 | 3.6 | 1 | 1 |
| ≥ population median | 180 212 | 3.0 | 0.85 [0.81-0.89] | 0.97 [0.92-1.02] |
| | | | | |
| *Weekly consumption of fish* | | | | |
| < population median | 118 348 | 3.2 | 1 | 1 |
| ≥ population median | 129 519 | 3.2 | 1.01 [0.97-1.06] | 1.01 [0.96-1.06] |

[1] during a median follow-up of 12.5 years
[2] hazard ratios (HR) [95%-CI] derived from a COX regression analysis including the following categorical and continuous independent variables: the five healthy diet components (binary), daily sleep duration (four levels), age, sex, ethnicity, smoking status, frequency of weekly alcohol intake, antidepressant use, assessment center region, BMI, systolic blood pressure, socioeconomic status, educational level, Insomnia symptoms frequency, and physical activity level.

© 2024 Nôga DA et al. *JAMA Network Open*.

**eTable 4. Association Between Short Sleep Duration and Incident Type 2 Diabetes Mellitus Stratified by Adherence to Consumption of Individual Food Groups**

| Exposure | Participants, n | T2DM cases[1], % | HR [95%-CI] | |
|---|---|---|---|---|
| | | | unadjusted | adjusted[2] |
| Sleep duration (hours/day) + Weekly consumption of red meat > population median | | | | |
| *7-8* | 61 375 | 3.6 | 1 | 1 |
| *6* | 15 810 | 4.1 | 1.15 [1.05-1.25] | 0.95 [0.97-1.04] |
| *5* | 3 209 | 5.5 | 1.56 [1.34-1.82] | 1.14 [0.97-1.33] |
| *3-4* | 654 | 9.3 | 2.62 [2.03-3.40] | 1.48 [1.14-1.93] |
| | | | | |
| Sleep duration (hours/day) + Weekly consumption of red meat ≤ population median | | | | |
| *7-8* | 125 900 | 2.7 | 1 | 1 |
| *6* | 33 212 | 3.4 | 1.27 [1.19-1.36] | 1.07 [0.99-1.14] |
| *5* | 6 445 | 4.4 | 1.68 [1.48-1.89] | 1.17 [1.03-1.33] |
| *3-4* | 1 262 | 6.1 | 2.36 [1.88-2.96] | 1.34 [1.07-1.69] |
| | | | | |
| Sleep duration (hours/day) + Weekly consumption of processed meat > population median | | | | |
| *7-8* | 114 273 | 3.3 | 1 | 1 |
| *6* | 29 807 | 3.9 | 1.18 [1.10-1.26] | 1.00 [0.94-1.07] |
| *5* | 5 586 | 5.5 | 1.68 [1.50-1.89] | 1.21 [1.08-1.37] |
| *3-4* | 1 131 | 7.6 | 2.34 [1.89-2.90] | 1.37 [1.10-1.70] |
| | | | | |
| Sleep duration (hours/day) + Weekly consumption of processed meat ≤ population median | | | | |
| *7-8* | 73 002 | 2.4 | 1 | 1 |
| *6* | 19 215 | 3.2 | 1.31 [1.20-1.44] | 1.07 [0.97-1.18] |
| *5* | 4 068 | 3.8 | 1.58 [1.34-1.87] | 1.06 [0.90-1.26] |
| *3-4* | 785 | 6.6 | 2.80 [2.13-3.70] | 1.49 [1.12-1.98] |
| | | | | |
| Sleep duration (hours/day) + Daily consumption of vegetables < population median | | | | |
| *7-8* | 65 277 | 3.0 | 1 | 1 |
| *6* | 17 889 | 3.8 | 1.29 [1.18-1.40] | 1.06 [0.97-1.16] |
| *5* | 3 480 | 5.2 | 1.75 [1.50-2.04] | 1.22 [1.04-1.43] |
| *3-4* | 732 | 6.8 | 2.35 [1.77-3.11] | 1.26 [0.94-1.67] |
| | | | | |
| Sleep duration (hours/day) + Daily consumption of vegetables ≥ population median | | | | |

© 2024 Nôga DA et al. *JAMA Network Open*.

| | | | | |
|---|---|---|---|---|
| *7-8* | 121 998 | 3.0 | 1 | 1 |
| *6* | 31 133 | 3.5 | 1.18 [1.10-1.27] | 1.00 [0.93-1.07] |
| *5* | 6 174 | 4.5 | 1.56 [1.38-1.76] | 1.13 [1.00-1.28] |
| *3-4* | 1 184 | 7.4 | 2.56 [2.07-3.17] | 1.51 [1.22-1.88] |
| | | | | |
| Sleep duration (hours/day) + Daily consumption of fruits < population median | | | | |
| *7-8* | 50 290 | 3.3 | 1 | 1 |
| *6* | 13 786 | 3.9 | 1.17 [1.06-1.29] | 0.98 [0.88-1.08] |
| *5* | 2 951 | 5.8 | 1.77 [1.51-2.07] | 1.29 [1.09-1.52] |
| *3-4* | 628 | 6.8 | 2.11 [1.56-2.86] | 1.29 [0.94-1.76] |
| | | | | |
| Sleep duration (hours/day) + Daily consumption of fruits ≥ population median | | | | |
| *7-8* | 136 985 | 2.8 | 1 | 1 |
| *6* | 35 236 | 3.5 | 1.24 [1.16-1.32] | 1.04 [0.97-1.11] |
| *5* | 6 703 | 4.3 | 1.55 [1.38-1.75] | 1.09 [0.97-1.24] |
| *3-4* | 1 288 | 7.4 | 2.65 [2.16-3.25] | 1.46 [1.18-1.80] |
| | | | | |
| Sleep duration (hours/day) + Weekly consumption of fish < population median | | | | |
| *7-8* | 88 892 | 2.9 | 1 | 1 |
| *6* | 23 963 | 3.7 | 1.28 [1.19-1.38] | 1.03 [0.95-1.11] |
| *5* | 4 573 | 5.1 | 1.80 [1.57-2.06] | 1.19 [1.04-1.37] |
| *3-4* | 920 | 7.1 | 2.50 [1.95-3.20] | 1.30 [1.01-1.67] |
| | | | | |
| Sleep duration (hours/day) + Weekly consumption of fish ≥ population median | | | | |
| *7-8* | 98 383 | 3.0 | 1 | 1 |
| *6* | 25 059 | 3.5 | 1.17 [1.08-1.27] | 1.02 [0.94-1.10] |
| *5* | 5 081 | 4.5 | 1.49 [1.30-1.70] | 1.14 [0.99-1.31] |
| *3-4* | 996 | 7.3 | 2.47 [1.95-3.12] | 1.53 [1.20-1.94] |

[1] during a median follow-up of 12.53 years

[2] hazard ratios (HR) [95%-CI] derived from a COX regression analysis including the following categorical and continuous independent variables: daily sleep duration (four levels), age, sex, ethnicity, smoking status, frequency of weekly alcohol intake, antidepressant use, assessment center region, BMI, systolic blood pressure, socioeconomic status, educational level, Insomnia symptoms frequency, and physical activity level.

© 2024 Nôga DA et al. *JAMA Network Open*.

**eFigure 1. Final Sample Estimation**

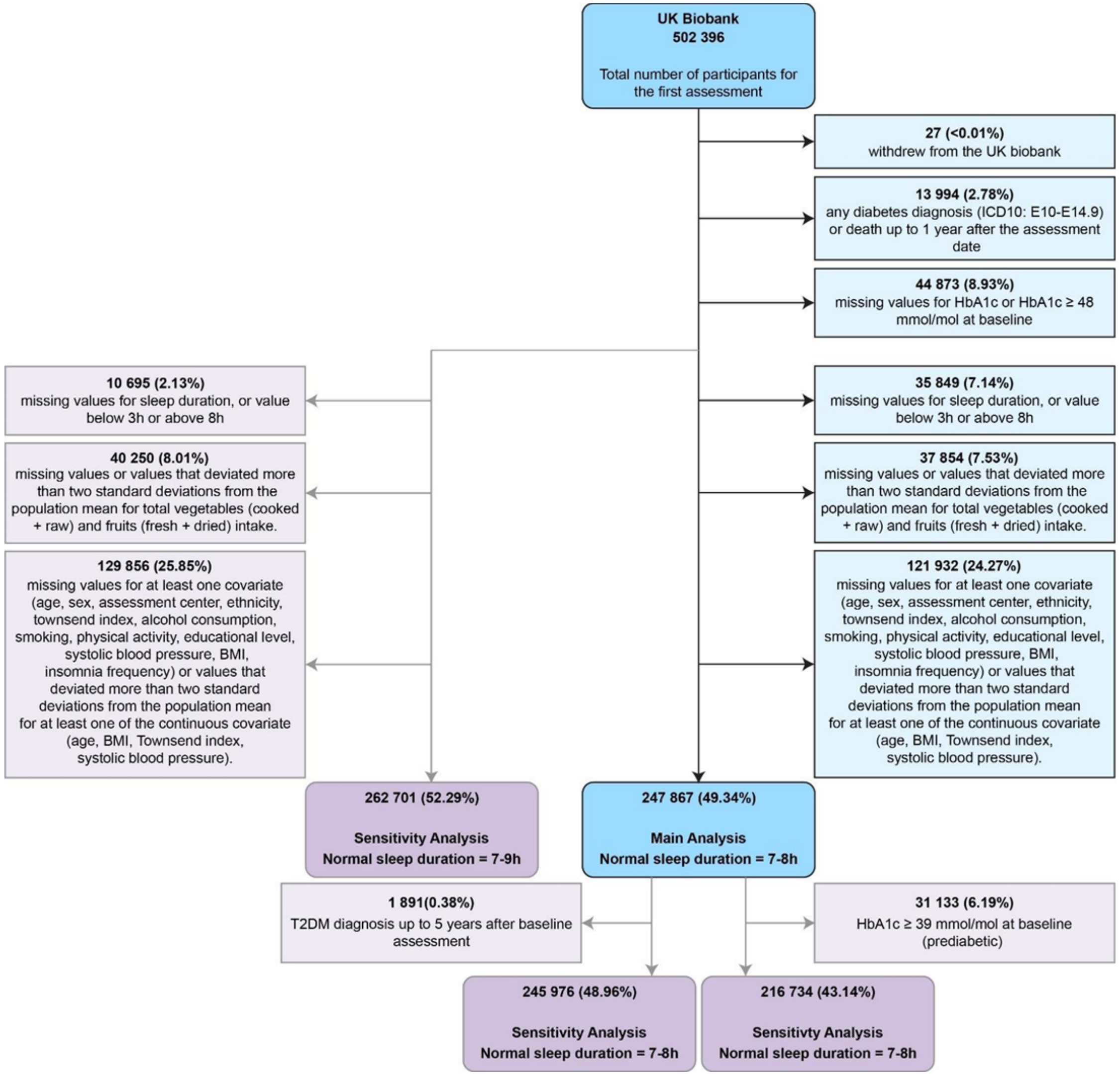


© 2024 Nôga DA et al. *JAMA Network Open*.

© 2024 Nôga DA et al. *JAMA Network Open*.

**eFigure 2. Association of Short Sleep Duration (Using 7-9 h of Daily Sleep as the Sleep Duration Reference Category) and Adherence to Healthy Diet With Incident Type 2 Diabetes Mellitus** (A) Unadjusted hazard ratios (HR) [95% CI] illustrating the link between sleep duration and incident type 2 diabetes mellitus (T2DM). (B) Unadjusted HR [95% CI] showcasing the relationship between healthy diet scores (HDS) and incident T2DM. (C) Adjusted HR [95% CI] presenting the association between sleep duration and incident T2DM. (D) Adjusted HR [95% CI] demonstrating the connection between HDS and incident T2DM.

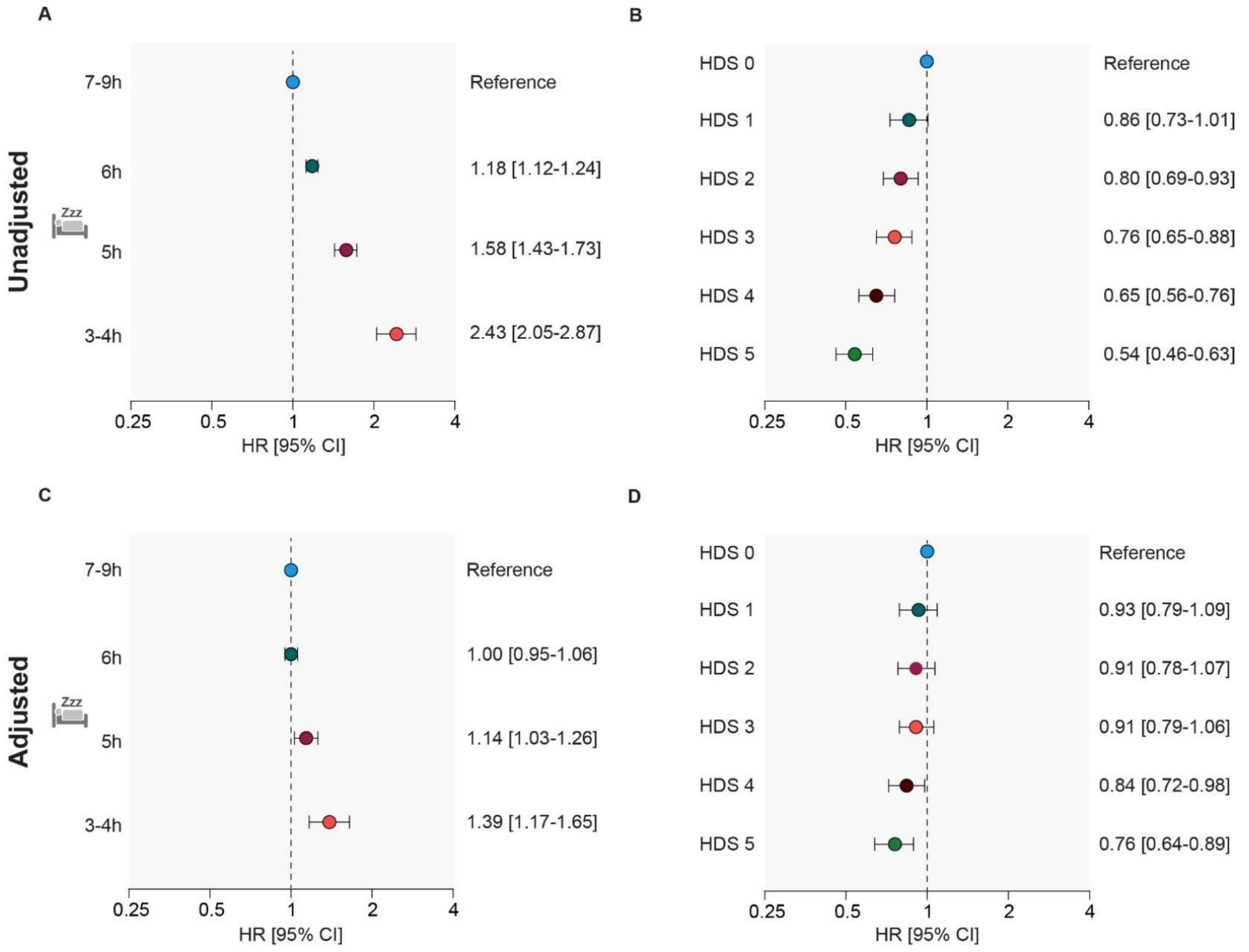


© 2024 Nôga DA et al. *JAMA Network Open*.

**eFigure 3. Association Between Short Sleep Duration (Using 7-9 h of Daily Sleep as the Sleep Duration Reference Category) and Incident Type 2 Diabetes Mellitus Stratified by Diet Status.** (A) Unadjusted Hazard Ratios HR [95% CI] illustrating the relationship between sleep duration and incident type 2 diabetes mellitus (T2DM) among participants with a T2DM- Non-Protective diet (B) Unadjusted HR [95% CI] illustrating the relationship between sleep duration and incident T2DM among participants with a T2DM-Protective diet. (C) Adjusted HR [95% CI] illustrating the relationship between sleep duration and incident T2DM among participants with a T2DM- Non-Protective diet. (D) Adjusted HR [95% CI] illustrating the relationship between sleep duration and incident T2DM among participants with a T2DM-Protective diet.

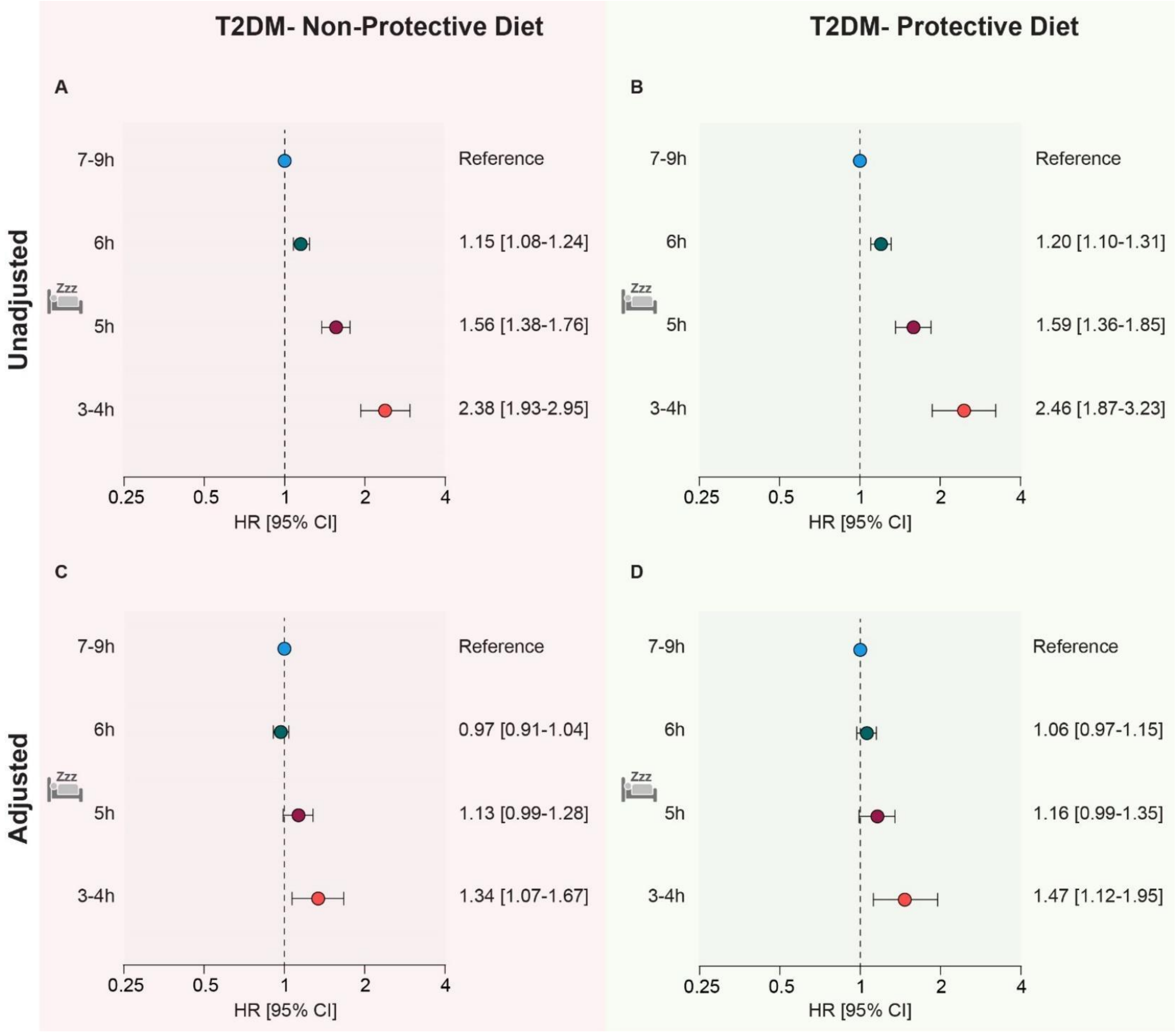


© 2024 Nôga DA et al. *JAMA Network Open*.

**eFigure 4. Association of Short Sleep Duration and Adherence to Healthy Diet With Incident Type 2 Diabetes Mellitus (Without First 5 Years T2DM Incidence)** (A) Unadjusted hazard ratios (HR) [95% CI] illustrating the link between sleep duration and incident type 2 diabetes mellitus (T2DM). (B) Unadjusted HR [95% CI] showcasing the relationship between healthy diet scores (HDS) and incident T2DM. (C) Adjusted HR [95% CI] presenting the association between sleep duration and incident T2DM. (D) Adjusted HR [95% CI] demonstrating the connection between HDS and incident T2DM.

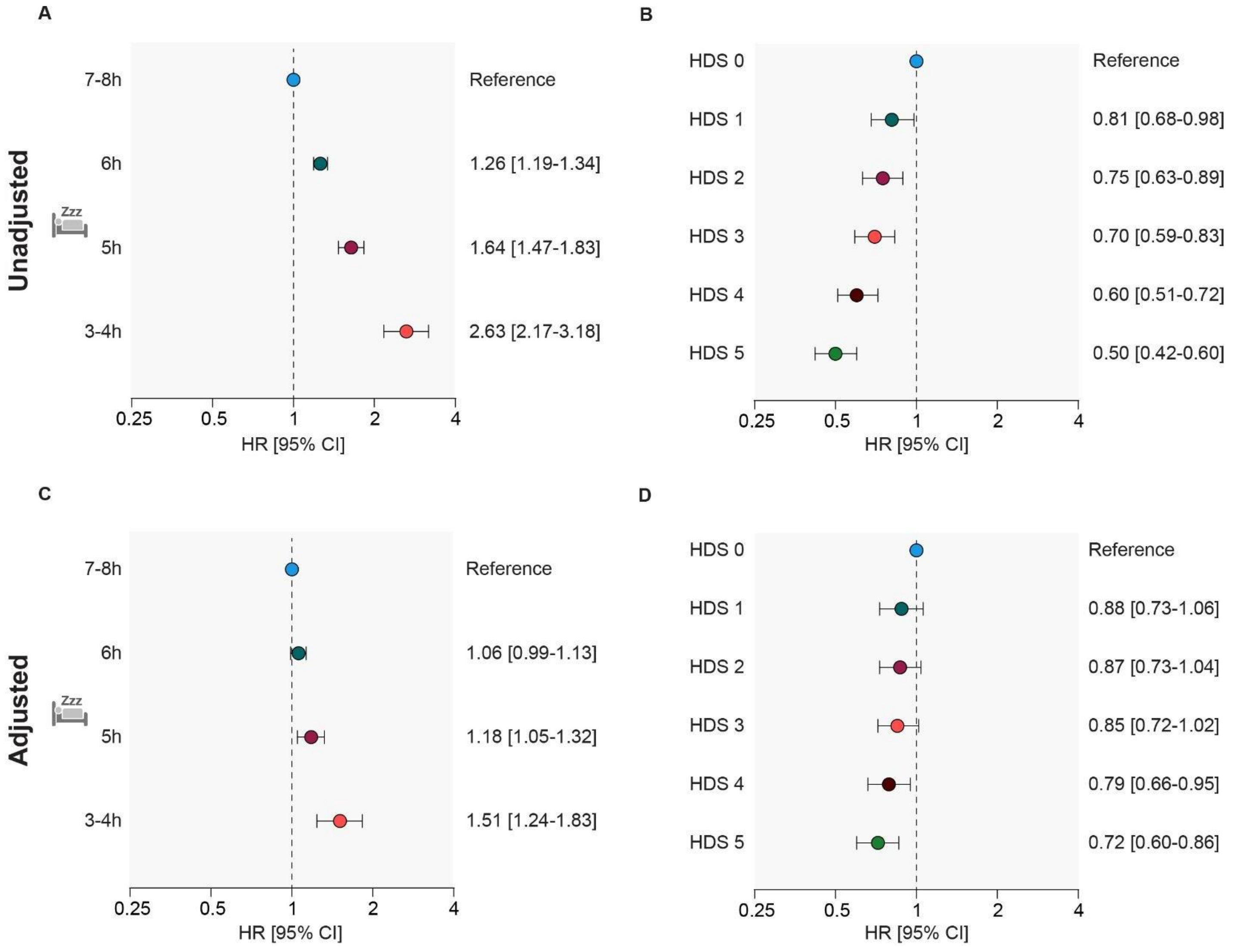


© 2024 Nôga DA et al. *JAMA Network Open*.

**eFigure 5. Association Between Short Sleep Duration and Incident Type 2 Diabetes Mellitus Stratified by Diet Status (Without First 5 Years T2DM Incidence).** (A) Unadjusted Hazard Ratios (HR) [95% CI] illustrating the relationship between sleep duration and incident type 2 diabetes mellitus (T2DM) among participants with a T2DM- Non-Protective diet (B) Unadjusted HR [95% CI] illustrating the relationship between sleep duration and incident T2DM among participants with a T2DM-Protective diet. (C) Adjusted HR [95% CI] illustrating the relationship between sleep duration and incident T2DM among participants with a T2DM- Non-Protective diet. (D) Adjusted HR [95% CI] illustrating the relationship between sleep duration and incident T2DM among participants with a T2DM-Protective diet.

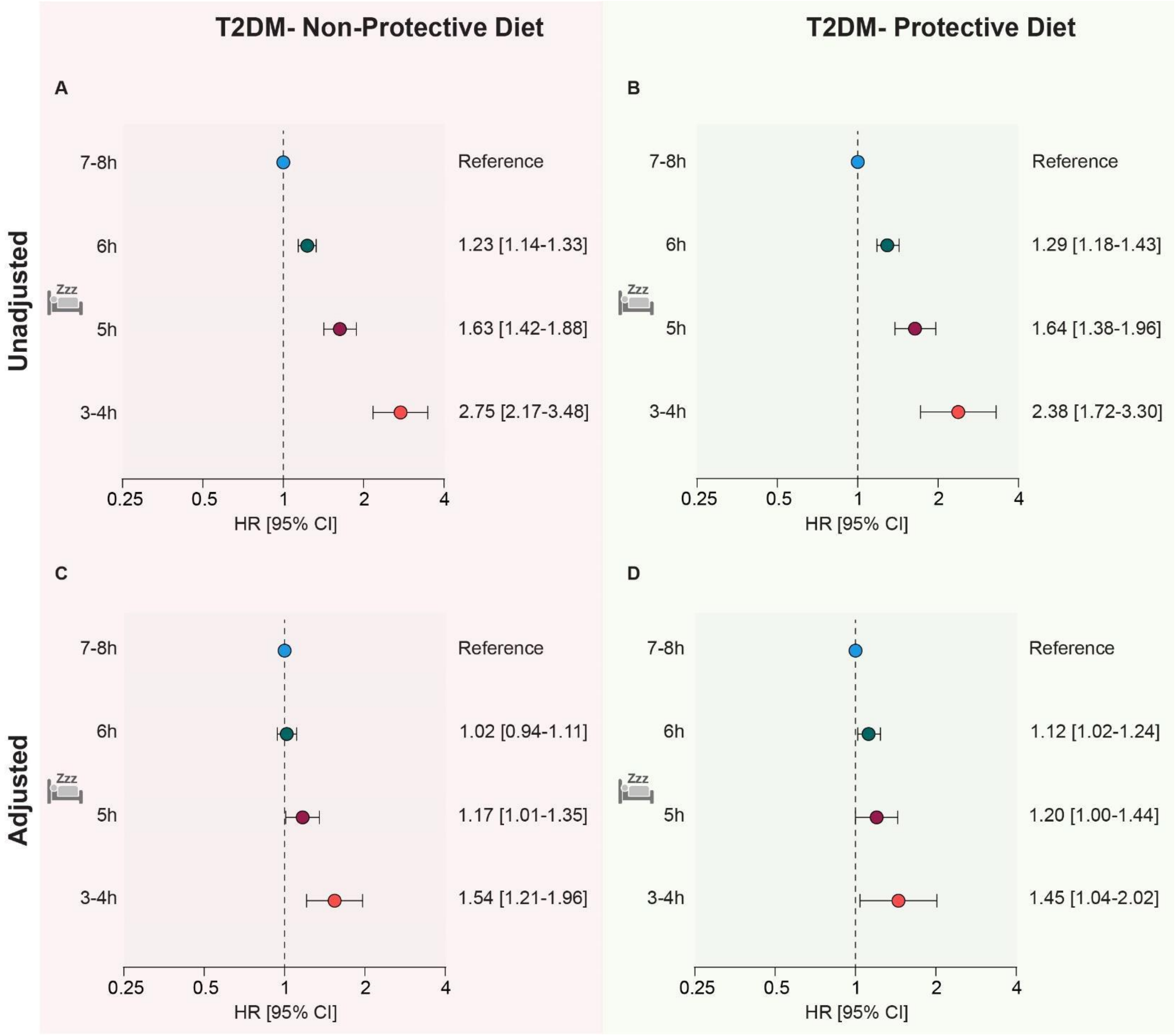


© 2024 Nôga DA et al. *JAMA Network Open*.

**eFigure 6. Association of Short Sleep Duration and Adherence to Healthy Diet With Incident Type 2 Diabetes Mellitus (Without Prediabetic Individuals).** (A) Unadjusted hazard ratios (HR) [95% CI] illustrating the link between sleep duration and incident type 2 diabetes mellitus (T2DM). (B) Unadjusted HR [95% CI] showcasing the relationship between healthy diet scores (HDS) and incident T2DM. (C) Adjusted HR [95% CI] presenting the association between sleep duration and incident T2DM. (D) Adjusted HR [95% CI] demonstrating the connection between HDS and incident T2DM.

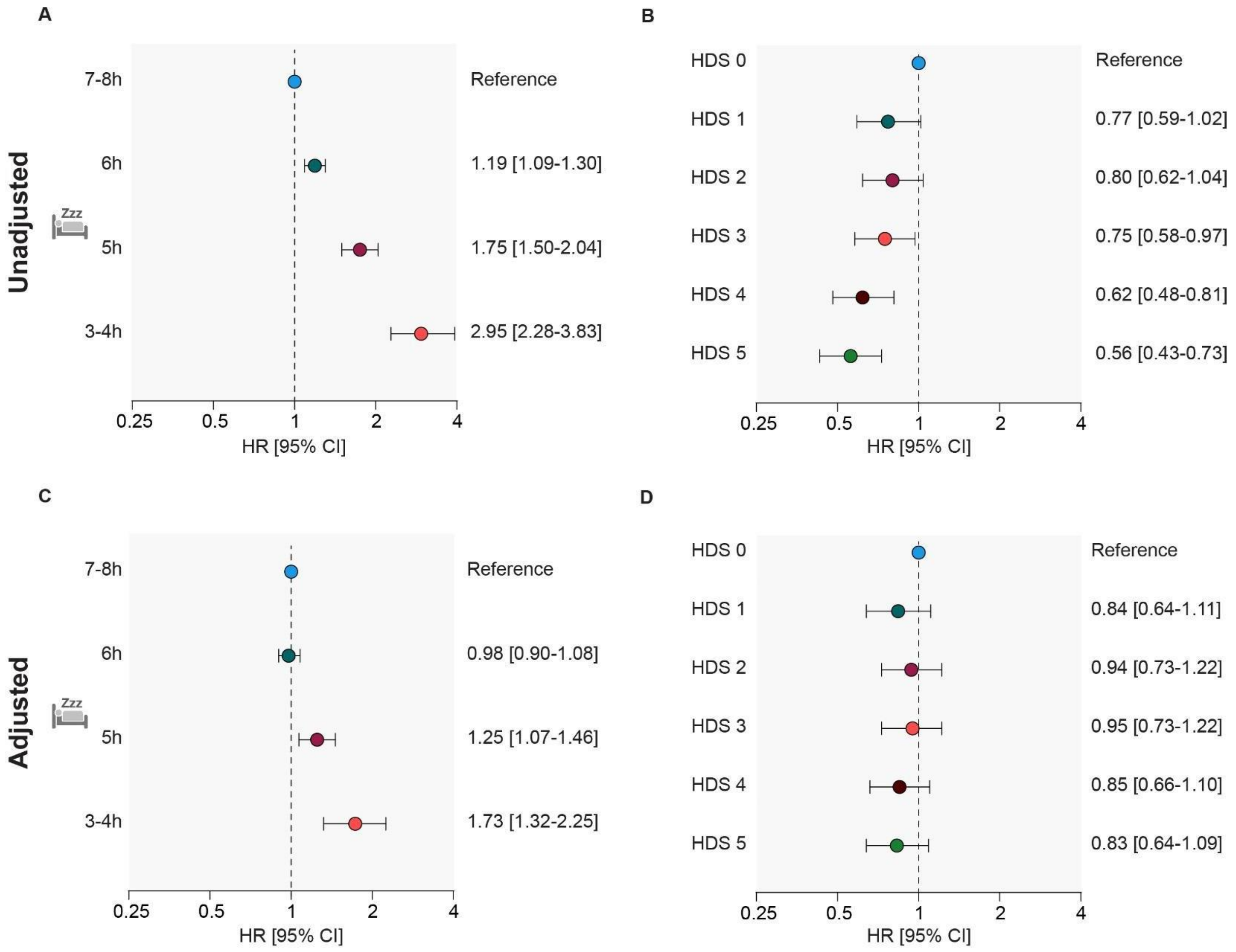


© 2024 Nôga DA et al. *JAMA Network Open*.

**eFigure 7. Association Between Short Sleep Duration and Incident Type 2 Diabetes Mellitus Stratified by Diet Status (Without Prediabetic Individuals).** (A) Unadjusted Hazard Ratios (HR) [95% CI] illustrating the relationship between sleep duration and incident type 2 diabetes mellitus (T2DM) among participants with a T2DM- Non-Protective diet (B) Unadjusted HR [95% CI] illustrating the relationship between sleep duration and incident T2DM among participants with a T2DM-Protective diet. (C) Adjusted HR [95% CI] illustrating the relationship between sleep duration and incident T2DM among participants with a T2DM- Non-Protective diet. (D) Adjusted HR [95% CI] illustrating the relationship between sleep duration and incident T2DM among participants with a T2DM-Protective diet.

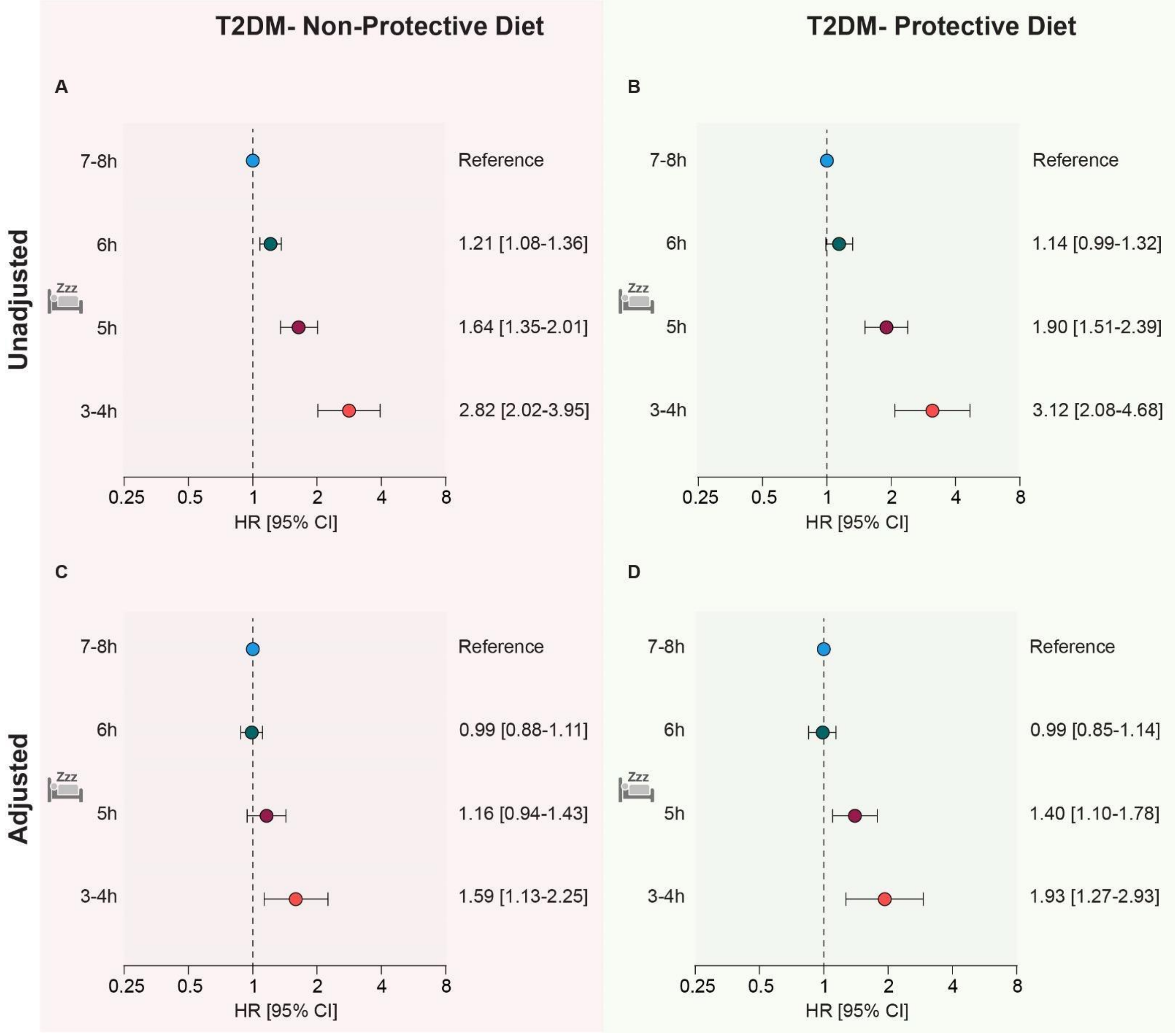


© 2024 Nôga DA et al. *JAMA Network Open*.
