# Supplementary material for: Habitual Short Sleep Duration, Diet, and Development of Type 2 Diabetes in Adults
Source: JAMA Netw Open. 2024 Mar 5;7(3):e241147. doi: 10.1001/jamanetworkopen.2024.1147 (PMC10915681; doi:10.1001/jamanetworkopen.2024.1147)
Supplement: Supplement 2. — Data Sharing Statement [file jamanetwopen-e241147-s002.pdf]

Data Sharing Statement

Nôga. Habitual Short Sleep Duration, Diet, and Development of Type 2 Diabetes in Adults. *JAMA Netw Open*. Published March 05, 2024. doi:10.1001/jamanetworkopen.2024.1147

Data
Data available: No

Additional Information
Explanation for why data not available: The data can be accessed upon approval from the UK Biobank.
